# From phenotype to mechanism: background-dependent function of *CtDnaJ.16* in thermotolerance-divergent *Coriolopsis trogii* haploids

**DOI:** 10.3389/fmicb.2026.1852348

**Published:** 2026-07-10

**Authors:** Lining Wang, Chaoxue Ma, Siyu Zhao, Jinjing Li, Lingjie Zhou, Laixin Dai, Biao Hu, Nianfang Ma, Qingfu Wang, Zhihai Huang

**Affiliations:** 1 Guangdong Engineering Laboratory of Biomass Value-Added Utilization, Guangdong Engineering Research & Development Center for Comprehensive Utilization of Plant Fiber, Guangzhou Key Laboratory for Comprehensive Utilization of Plant Fiber, Institute of Biological and Medical Engineering, Guangdong Academy of Sciences, Guangzhou, China; 2 The Second Clinical College, Guangzhou University of Chinese Medicine, Guangzhou, China

**Keywords:** *Coriolopsis trogii*, DnaJ, haploid-specific, high-temperature, trait

## Abstract

The two haploids, Ct001_29 and Ct001_31, derived from the thermotolerant strain *Coriolopsis trogii* Ct001, differ in mating type and exhibit distinct thermotolerance capacities. To investigate the underlying molecular mechanisms, their phenotypic responses to different temperatures were characterized, and comparative RNA-sequencing (RNA-Seq) analyses were performed using an upgraded, manually curated genome annotation. Phenotypic observations revealed that Ct001_31 displayed more robust hyphal growth at 35 °C than Ct001_29, including denser mycelia, larger hyphal diameter, smoother hyphal surfaces, and the ability to sustain growth at 40 °C. Through manual correction, a high-quality genome annotation for Ct001_31 was generated, comprising 12,953 high-confidence genes. Comparative RNA-Seq analysis identified 108 up-regulated and 121 down-regulated genes commonly shared between the two haploids at 35 °C. The up-regulated genes in Ct001_29 were significantly enriched in functions related to protein folding and heat response, whereas no significant functional enrichment was observed among the differentially expressed genes (DEGs) in Ct001_31 at 35 °C. Notably, *CtDnaJ.16* showed increased expression in Ct001_31 at 35 °C, while no significant change was observed in Ct001_29. Functional investigation revealed that *Ct31DnaJ.16* was specifically involved in thermotolerance in Ct001_31, with transformants overexpressing this gene capable of germinating at 43 °C. In contrast, neither overexpression nor RNA interference of *Ct29DnaJ.16* in Ct001_29 led to detectable phenotypic alterations. These findings demonstrate that *CtDnaJ.16* plays a strikingly haploid-specific role in thermotolerance, deepening our understanding of functional differentiation between allelic genes.

## 1 Introduction

High temperatures induce the accumulation of reactive oxygen species, including hydrogen peroxide (H_2_O_2_) and superoxide anions, thereby triggering oxidative stress that impairs cellular functions (Luo et al., 2021). For macrofungi, which predominantly thrive under moderate temperature conditions, heat stress represents a major environmental constraint (Luo et al., 2021). Most macrofungi have relatively low optimal growth temperatures, and exposure to ambient temperatures exceeding 25 °C typically inhibits both mycelial growth and fruiting body development, severely compromising yield and quality (Sakamoto, 2018; Liu Q. L. et al., 2020). This thermal sensitivity not only poses challenges to industrial cultivation but is further exacerbated by the increasing frequency of extreme weather events, highlighting the urgent need to elucidate the molecular mechanisms underlying fungal thermotolerance.

*Coriolopsis trogii* presents a valuable model for investigating thermotolerance mechanisms. Its mycelium exhibits an optimal growth temperature near 35 °C, significantly higher than that of most filamentous fungi (Wang et al., 2021). Previous researches on heat tolerance in macrofungi have largely focused on dikaryotic strains (Liu et al., 2024; Zhou D. X. et al., 2024), in which phenotypic traits arise from the combined effect of two genetically distinct nuclei, thereby complicating the dissection of underlying molecular pathways. A recent investigation into heat-responsive mechanisms in compatible haploids of *Lentinula edodes* highlights the advantages of using haploid strains (Guo et al., 2025), whose simpler genetic backgrounds enables a more direct link between genotype and phenotype. To fully leverage this advantage, a high-quality genome annotation is essential.

Molecular chaperones, particularly the HSP70/HSP40 system, play a central role in the heat shock response by facilitating protein folding, translocation, and complex assembly (Liu X. B. et al., 2020). DnaJ (HSP40) proteins are defined by a conserved J domain that mediates their functional interaction with HSP70 partners (Pellecchia et al., 1996). Although overexpression of *DnaJ* has been shown to enhance thermotolerance in macrofungi, such as *L. edodes* (Wang et al., 2020), their functional role in *C. trogii* remains entirely unexplored.

In this study, the molecular basis of heat adaptation was investigated using two sexually compatible haploids, Ct001_29 and Ct001_31, both derived from the thermotolerant *C. trogii* strain Ct001 (Wang et al., 2021). To establish a reliable genomic foundation, the genome annotation of Ct001_31 was upgraded through manual, gene-by-gene curation. Leveraging this improved resource, comparative phenotypic and RNA-Seq analyses were conducted on the two haploids under different temperatures. These analyses identified a candidate *DnaJ* gene, *CtDnaJ.16*, which exhibited divergent expression patterns between the haploids. Functional validation via genetic transformation subsequently revealed that *CtDnaJ.16* plays a strikingly haploid-specific role in thermotolerance, providing new insights into the mechanisms underlying heat adaptation in macrofungi.

## 2 Materials and methods

### 2.1 Strain cultivation and phenotypic traits assay

The haploid strains Ct001_29 and Ct001_31 were maintained on potato dextrose agar (PDA) at 4 °C. Fresh mycelia were obtained by subculturing the haploids on PDA at 25 °C for two successive generations. For solid culture, a single mycelial block (5 mm in diameter) from each haploid was inoculated on PDA plates (90 mm in diameter), with or without an overlaid cellophane, and cultured at 25 °C and 35 °C, respectively. For liquid culture, six mycelial blocks per haploid were inoculated into 100 mL of potato dextrose broth (PDB) and cultured at 25 °C and 35 °C with shaking at 150 rpm. To determine biomass accumulation on solid media, mycelia were collected from five plates per condition and dried at 60 °C to constant weight. For liquid media, mycelia from each 100 mL culture were harvested by gauze filtration after removal of the initial inoculation blocks, washed with sterile water, and dried at 60 °C to constant weight. All measurements were performed in triplicate.

To observe the microscopic surface morphology of the haploids, mycelia cultured on PDA at 25 °C and 35 °C were scraped off using a sterile spoon. The samples were then fixed with 2.5% glutaraldehyde and 1% osmium acid, dehydrated through a graded ethanol series, and dried using a critical point dryer (EM CPD300, Leica Microsystems, Germany). After sputter-coating with gold, the samples were imaged using a SU8010 Ultra-High Resolution (1.0 nm) Scanning Electron Microscope (Hitachi, Ltd., Japan).

Nitroblue tetrazolium (NBT) and 3,3′-diaminobenzidine (DAB) staining were employed to determine superoxide anion and H_2_O_2_ levels, respectively. For DAB staining, mycelial plates were treated with 8 mL of freshly prepared DAB solution (1 mg/mL, pH 3.8) and incubated at room temperature for 12 h. For NBT staining, plates were similarly treated with 8 mL of freshly prepared 0.1% (w/v) NBT solution in 1 × PBS and then incubated at room temperature for 2 h.

Contact angle measurement was performed using an OCA Goniometer (DataPhysics Instruments GmbH, Germany) to assess the hydrophobicity and wetting properties of the mycelial surface.

### 2.2 Genome annotation update

The genome annotation of Ct001_31 was manually curated using Apollo (Dunn et al., 2019), guided by alignments of RNA-Seq data derived from diverse conditions-including different carbon sources, temperatures, and different developmental stages-to the reference genome sequence (Wang et al., 2021). Gene boundaries and splice sites were refined through a series of manual operations, including deletion, merging, splitting, creation, extension, and retraction. The annotation versions before and after manual curation were compared using Gffcompare v0.10.4.^1^ Protein-coding genes were functionally annotated by querying the following databases: Pfam (El-Gebali et al., 2019), UniProt,^2^ eggNOG (Huerta Cepas et al., 2019) and InterProScan (Jones et al., 2014). The completeness of the annotation was evaluated by Benchmarking Universal Single-Copy Ortholog (BUSCO) analysis (Simão et al., 2015) with the odb10 database.

### 2.3 RNA-Seq of mycelia cultured at different temperatures

Mycelia of Ct001_29 and Ct001_31 were cultured on PDA at either 25 °C or 35 °C for 5 days. For each sample, mycelia were harvested from 10 plates and pooled as one replicate. Four replicates were established for Ct001_29 at 35 °C, while three replicates were prepared for the remaining treatments. Total RNA was extracted from all collected samples as previously described (Wang et al., 2018). Briefly, frozen samples ground in liquid nitrogen were processed using an OMEGA RNA kit (R6827-01) following the manufacturer’s instructions, and only RNA samples with an Integrity Number of 7.5 or higher were retained. RNA-Seq libraries were generated in accordance with MGI sequencing platform guidelines, and each library was sequenced to obtain a minimum of six Gb of 150-bp paired-end reads.

### 2.4 Gene expression analysis

RNA-Seq data analysis was performed according to the method described by Li (Li, 2019). Raw RNA-Seq reads were trimmed and quality-filtered using Skewer (Jiang et al., 2014). Clean reds were aligned to Ct001_31 genome using HISAT2, and expression levels were quantified as transcript per million (TPM) with StringTie (Pertea et al., 2016). Gene expression patterns were visualized by hierarchical clustering of TPM values using the R package ‘pheatmap’.^3^ Differential expression analysis was performed with DESeq2 (Wang et al., 2010) under two comparison schemes: (1) within the same haploid across different temperatures, and (2) between the two haploids at the same temperature. Genes exhibiting a |log_2_fold change| ≥ 1, and an adjusted *p* ≤ 1e-3 were defined as DEGs.

### 2.5 Identification and analysis of the *DnaJ* and *HSP70* gene families in *C. trogii*

Genes containing a complete DnaJ domain (Pfam ID: PF00226) in either the Ct001_29 or Ct001_31 genome were designated as members of the *DnaJ* family. All DnaJ proteins identified in Ct001_31 were compared with those from Ct001_29 using BlastP (Camacho et al., 2009), and protein sequences sharing ≥85% identity were considered allelic pairs. The same approach was applied to identify *HSP70* family (Pfam ID: PF00012) members. Subcellular localization prediction was conducted using DeepLoc 2.1.^4^ Promoter sequences were defined as the 2000 bp region upstream of transcription start site. Minimap2 (Li, 2018) was employed to perform sequence alignment between the promoter of *Ct29DnaJ.16* and that of *Ct31DnaJ.16*. Bcftools^5^ was used to identify genetic variants. The JASPAR database^6^ was used to predict candidate cis-elements within promoter regions, while the Integrative Genomics Viewer (Thorvaldsdóttir et al., 2013) was applied for concurrent visualization of genetic variants and cis-elements.

### 2.6 Gene functional enrichment analysis

Functional enrichment of Pfam domains and Gene Ontology (GO) terms was performed using clusterProfiler (Yu et al., 2012), and enrichment results with *p*-value < 1e-3 were retained.

### 2.7 Gene expression validation by quantitative real-time PCR (qPCR)

The RNA samples for RNA-Seq were reverse-transcribed to synthesize cDNA, which was subsequently used for qPCR validation. qPCR reactions were carried out using ChamQ Universal SYBR qPCR Master Mix (Vazyme, Nanjing, China) following a previously established protocol (Wang et al., 2025). Glyceraldehyde 3-phosphate dehydrogenase (*GAPDH*) or ubiquitin-conjugating enzyme (Tt31c28g0121441) served as the internal reference gene. All primers, detailed in Supplementary Table S1, were designed and commercially synthesized by Guangzhou IGE Biotechnology Co., Ltd. (Guangzhou, China).

### 2.8 Validation of genomic features: gene structure, non-canonical splicing, and genetic variations

Primer sets were designed for three validation purposes: (1) amplification of the coding sequences (CDS) of three *GH* and six *DnaJ* genes using primers located in the untranslated regions (UTR); (2) amplification of 11 non-canonical splicing sites representing five types (GC-AG, AT-AC, GT-AA, GT-GT, GA-AC) using primers flanking the splice junctions; and (3) amplification of 48 single nucleotide polymorphisms (SNPs) and seven insertions/deletions (Indels) using primers targeting conserved flanking regions. All primer sequences were listed in Supplementary Tables S2, S3. The PCR amplification was performed in a 20 μL reaction mixture containing 1 μL of template, 10 μL of 2 × Taq Master Mix (Vazyme Biotech Co., Ltd), 0.4 μL each of forward and reverse primers (10 μmol/L), and 8.2 μL of ddH_2_O. The thermal cycling included an initial denaturation at 95 °C for 3 min; followed by 32 cycles of 95 °C for 30 s, 55–60 °C for 30 s, and 72 °C for 30–90 s; and a final extension at 72 °C for 5 min. Templates were selected based on experimental aims: for amplifying CDS regions, cDNA from Ct001_31 served as the template; for validating non-canonical splicing sites, both gDNA and cDNA from sample Ct001_31 were used; and for detecting genetic variations, gDNA from Ct001_29 and Ct001_31 was employed. All PCR amplification products were analyzed via agarose gel electrophoresis, and the Sanger sequencing was conducted by Guangzhou IGE Biotechnology Co., Ltd. (Guangzhou, China).

### 2.9 Construction of overexpression (OE) and RNA interference (RNAi) plasmids and acquisition of transformants

The CDS sequences of *Ct31DnaJ.16* and *Ct29DnaJ.16* were synthesized by Guangzhou IGE Biotechnology Co., Ltd. (Guangzhou, China) and cloned into modified fungal OE and RNAi vectors (Hou et al., 2019), respectively. For RNAi constructs, sequences encoding short hairpin RNAs (shRNAs) targeting the respective *CtDnaJ.16* alleles were designed and inserted into the RNAi vector. The resulting plasmids, OE-*Ct31DnaJ.16*, OE-*Ct29DnaJ.16*, RNAi-*Ct31DnaJ.16*, and RNAi-*Ct29DnaJ.16*, were propagated in TOP10 cells and then introduced into *Agrobacterium tumefaciens* GV3101, respectively. Fungal transformation of Ct001_29 or Ct001_31 was performed following an established *Agrobacterium*-mediated method (Hou et al., 2019). Briefly, young mycelia were co-cultivated with an *Agrobacterium* suspension carrying the target plasmid in the presence of acetosyringone. Transformants were selected on PDA plates supplemented with hygromycin B (0.07 mg/mL for Ct001_29, or 0.16 mg/mL for Ct001_31) and 0.3 mg/mL cefotaxime, and confirmed by PCR amplification of the hygromycin resistance gene. For phenotypic evaluation, confirmed transformants and wild-type (WT) were inoculated on PDA plates and incubated at 25 °C, 35 °C, 40 °C, and 43 °C to assess thermotolerance. DAB and NBT staining of transformants and gene expression assays followed the procedures in Sections 2.1 and 2.7, respectively.

### 2.10 Yeast two-hybrid (Y2H) assays

The full-length CDS sequence of *Ct31DnaJ.16* was cloned into the pGBKT7 (bait) vector between *Sfi*I and *Not*I sites via homologous recombination. Each of the seven *CtHSP70* genes (*CtHSP70.1* to *CtHSP70.7*) was individually inserted into the pGADT7 (prey) vector (Supplementary Figure S1). All primers are listed in Supplementary Table S4. To test for protein–protein interactions, each pGADT7-*CtHSP70* prey plasmid was co-transformed into the Y2HGold yeast strain along with the pGBKT7-*Ct31DnaJ.16* bait plasmid. Transformants were first selected on SD/−Trp/−Leu medium to verify the presence of both plasmids. Interaction assays were then performed by spotting the co-transformants on SD/−Trp/−Leu/-His/−Ade medium supplemented with Aureobasidin A (+AbA), with or without prior heat stress treatment at 37 °C for 1 h. Control strains were included in parallel: Y2HGold[pGBKT7-53 + pGADT7-T] as a positive control, Y2HGold[pGBKT7-Lam + pGADT7-T] as a negative control, and Y2HGold[pGBKT7-*Ct31DnaJ.16* + pGADT7] to test for bait autoactivation.

### 2.11 Statistical analysis

Statistical significance between two groups was assessed using Welch’s t-test. For comparisons involving three or more groups, one-way analysis of variance (ANOVA) was performed. When ANOVA indicated significant differences, Duncan’s post-hoc test was applied for multiple comparisons. A significance level of *p*-value < 0.05 was used for all statistical analysis.

## 3 Results

### 3.1 Phenotypic differences between two haploids cultured at different temperatures

**Figure 1:**
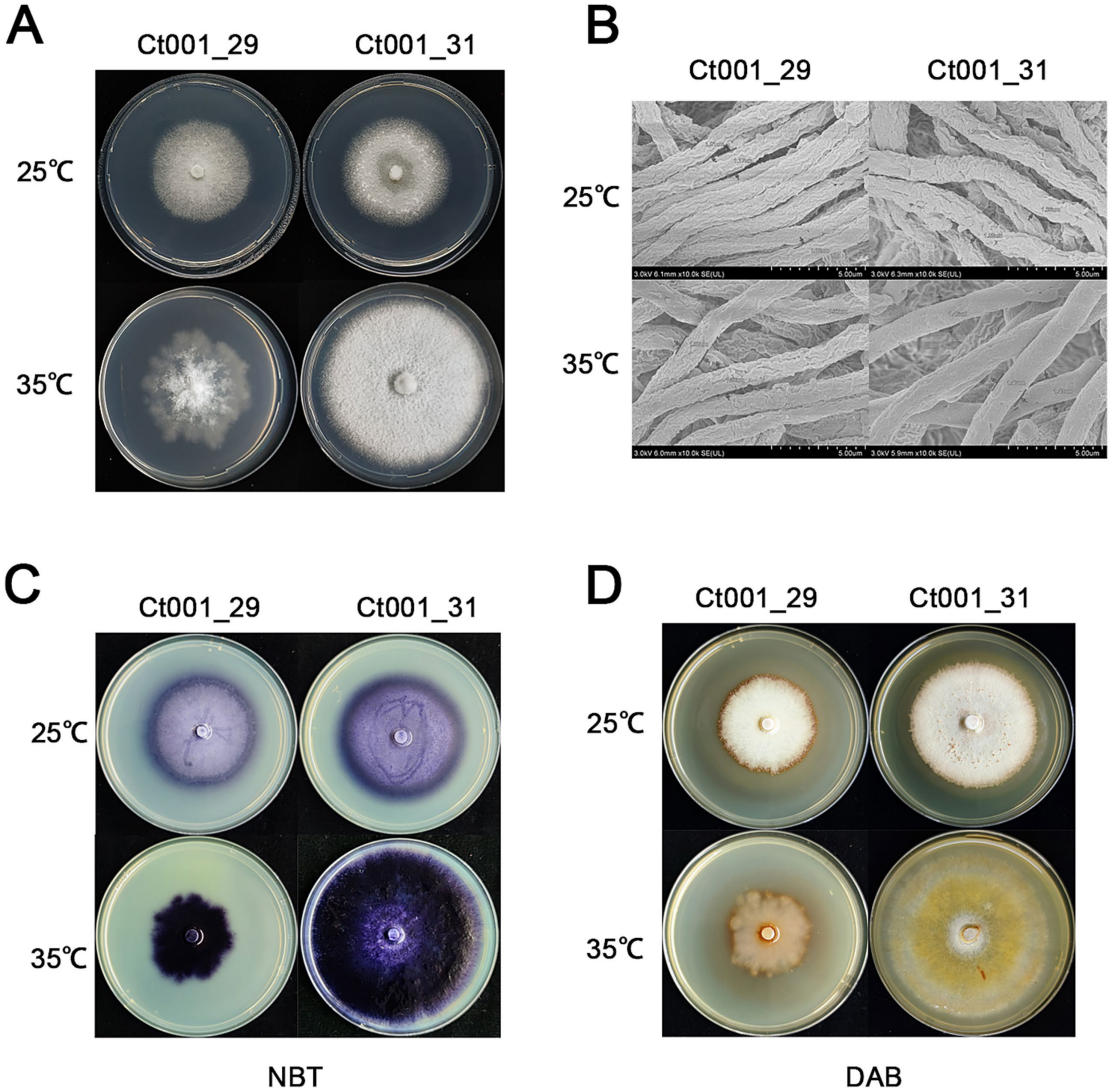
Physiological traits of Ct001_29 and Ct001_31 cultured at 25 °C and 35 °C. **(A)** Mycelial morphology. **(B)** SEM images of mycelia. **(C)** Superoxide anion levels detected by NBT staining. **(D)** H_2_O_2_ content revealed by DAB staining.

Ct001 is a thermotolerant strain with an optimal growth temperature of 35 °C. Although both mating-compatible haploids, Ct001_29 and Ct001_31, grew faster at 35 °C than at 25 °C, Ct001_29 failed to germinate at 40 °C (Figure 1A; Supplementary Figure S2). At 35 °C, Ct001_29 produced sparse, prostrate peripheral hyphae with irregular tips, while the central region formed dense, pure-white hyphae (Figure 1A). In contrast, under the same conditions, Ct001_31 developed dense aerial hyphae featuring neatly aligned tips (Figure 1A). Notably, hyphae of both haploids showed stronger hydrophobicity when cultured at 25 °C, whereas those grown at 35 °C were completely non-hydrophobic (Supplementary Figure S2). The biomass of liquid-cultured mycelia was significantly higher in Ct001_31 than in Ct001_29 at both 25 °C and 35 °C, though no significant difference was observed for mycelia grown on solid medium at 25 °C (Supplementary Figure S2). Scanning electron microscopy (SEM) analysis revealed distinct microstructural traits between the two haploids (Figure 1B). At 35 °C, the hyphal diameter of Ct001_29 was larger than at 25 °C, and Ct001_31 exhibited an even greater hyphal diameter than Ct001_29 (Supplementary Figure S2). At 35 °C, the hyphal surface of Ct001_31 appeared smooth (Figure 1B). *In situ* staining revealed that at 25 °C, the peripheral hyphae of Ct001_29 and Ct001_31 exhibited higher levels of superoxide anions and H_2_O_2_ (Figures 1C,D). In contrast, hyphae cultured at 35 °C showed universally higher superoxide anion and H₂O₂ content across both central and peripheral regions (Figures 1C,D). Together, these findings indicate that while the two haploids share certain physiological responses to temperature, they also display haploid-specific structural and biochemical adaptations under identical thermal conditions.

### 3.2 Updating of the annotation of Ct001_31 genome

**Figure 2:**
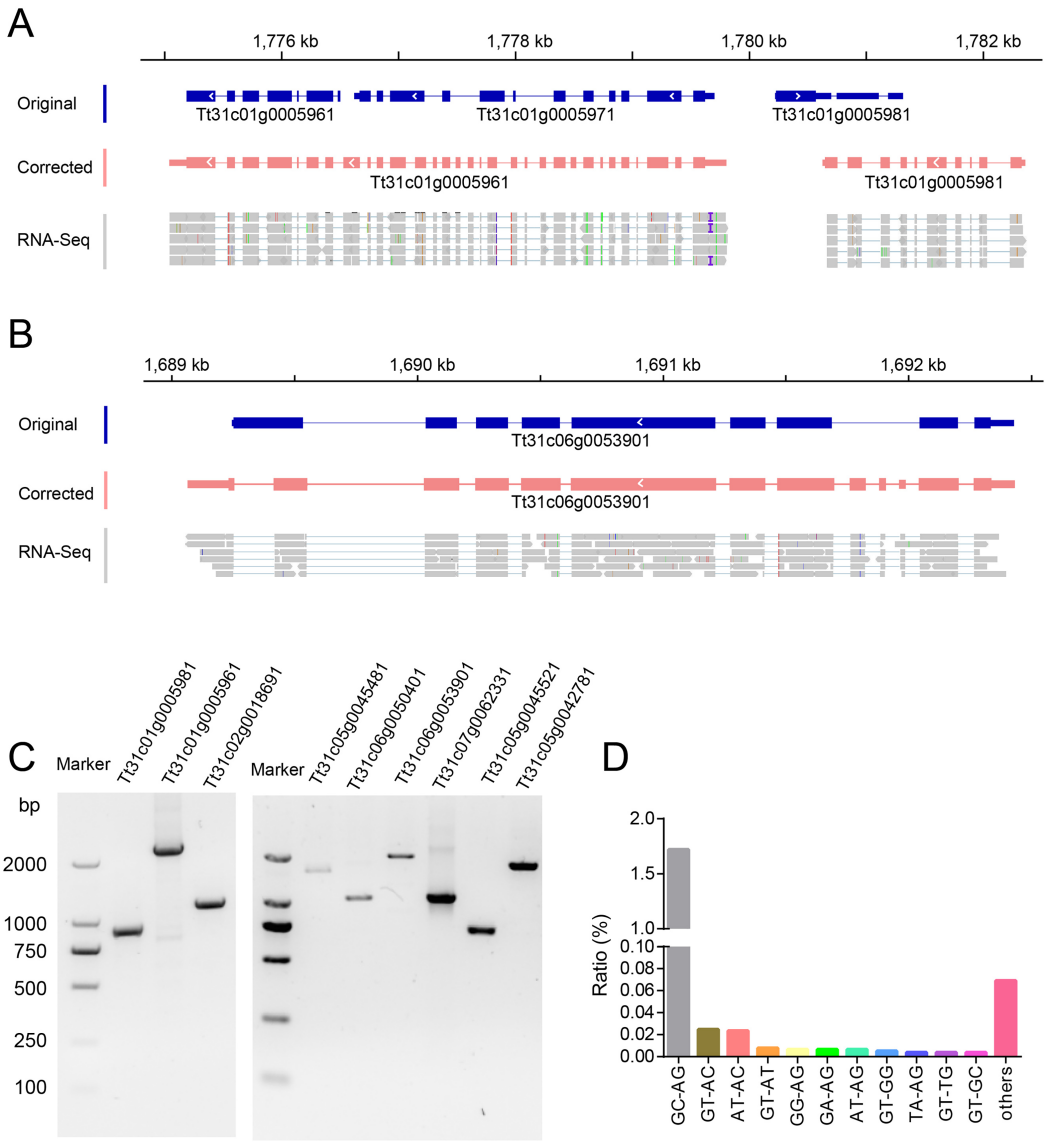
Updating the annotation of the Ct001_31 genome. **(A)** Comparison of gene structures of two *GH* genes before and after manual correction. **(B)** Comparison of gene structures of one *DnaJ* gene before and after manual correction. Arrows indicate transcription direction. **(C)** Electrophoresis of PCR amplification products from selected *GH* and *DnaJ* genes. **(D)** Distribution of non-canonical splice sites.

Guided by RNA-Seq transcript data, the gene structures of all predicted gene models in Ct001_31 were manually corrected. A total of 5,855 full-match genes remained unchanged, 1,158 genes were deleted, and 674 genes were newly added. During this process, 7,280 previously annotated exons were deleted, 6,537 novel exons were added, 6,784 introns were deleted, and 8,112 novel introns were added. Following manual curation, 12,953 high-confidence genes were annotated in the Ct001_31 genome. Manual correction revealed substantial inaccuracies in the tool-predicted gene structures, even for well-characterized gene families. For example, genes encoding glycoside hydrolase (GHs), previously reported to be significantly expanded in this strain (Wang et al., 2021), and DnaJ proteins were extensively revised. Specifically, one *GH* gene was reconstructed by combining two separate genes and adding five introns (Tt31c01g0005961), and a previously unrecognized *GH* gene (Tt31c01g0005981) was rescued by creating a new gene adjacent to it (Figure 2A). Another *GH* gene (Tt31c05g0039571) was rescued by adding seven exons and seven introns into its endogenous locus. In addition, the 3′ region of a *GH* gene was extended with two newly identified introns and exons (Tt31c02g0018691), and the 5′ region of another *GH* gene was completed with three additional introns and three exons (Tt31c02g0029931a; Supplementary Figure S3). Among the *DnaJ* family, one gene (Tt31c06g0053901) was corrected by adding three exons and three introns near 3′ region and one intron and one exon at the 5′ region (Figure 2B). The remaining five *DnaJ* genes underwent various corrections, including the addition of exons or introns, shortening or lengthening of UTRs, merging of adjacent genes, and splitting of a single gene into two separate genes (Supplementary Figure S3). The manually corrected structures of *GH* and *DnaJ* genes were confirmed by PCR amplification and Sanger sequencing (Figure 2C).

The corrected gene sets were evaluated at multiple levels. Following manual curation, BUSCO analysis indicated genome completeness ranging from 99.57% (polyporales_odb10) to 99.76% (agaricomycetes_odb10; Supplementary Table S5). Missing and fragment genes were examined individually and found to lack supporting transcriptomic evidence. The proportion of functionally annotated genes increased substantially in the corrected gene sets. Specifically, approximately 78.37% of the genes were annotated using EggNOG, 61.21% using Pfam, 69.60% using InterProScan, and 64.56% using UniProt. To further validate the completeness of the manually corrected gene structures, transcriptomes were individually assembled *de novo* using Trinity. The assembled transcripts were then aligned to the corrected mRNA sequences, revealing an increase in the average transcript coverage from 77.70 to 83.52%. These results demonstrate that the corrected gene structures are more complete, offer improved representation of transcriptomic data, and are expected to enhance the accuracy of gene expression quantification.

In addition, manual curation revealed abundant non-canonical splicing sites in the Ct001_31 genome. A total of 1,444 non-canonical splice sites were identified, with GC-AG being the most abundant (1.7202%), followed by GT-AC (0.0248%), and AT-AC (0.0235%; Figure 2D). Several non-canonical splice sites were randomly selected for experimental validation, and the results confirmed the accuracy of these non-canonical introns (Supplementary Figure S4).

### 3.3 Ct001_29 and Ct001_31 displayed different response to high temperature

To investigate the transcriptional responses of the two haploids to elevated temperature, RNA-Seq on mycelia of Ct001_29 and Ct001_31 after 5 days of culture at 25 °C and 35 °C were performed. The sequencing data were of sufficient depth and quality for downstream analysis (Supplementary Table S6). Cluster analysis of genome-wide gene expression patterns showed that transcriptional differences between the two haploids were more pronounced than those induced by temperature variation (Supplementary Figure S5). Using the manually curated “golden genome annotation” described above, we performed pairwise comparisons of RNA-Seq data, both between temperatures within each haploid and between the two haploids at the same temperature (Supplementary Figure S6). At 35 °C, Venn analysis of DEGs revealed 108 commonly up-regulated and 121 commonly down-regulated genes in both haploids. Ct001_29 displayed 780 uniquely up-regulated and 678 uniquely down-regulated DEGs, whereas Ct001_31 had 599 uniquely up-regulated and 807 uniquely down-regulated DEGs (Supplementary Figure S7A).

**Figure 3:**
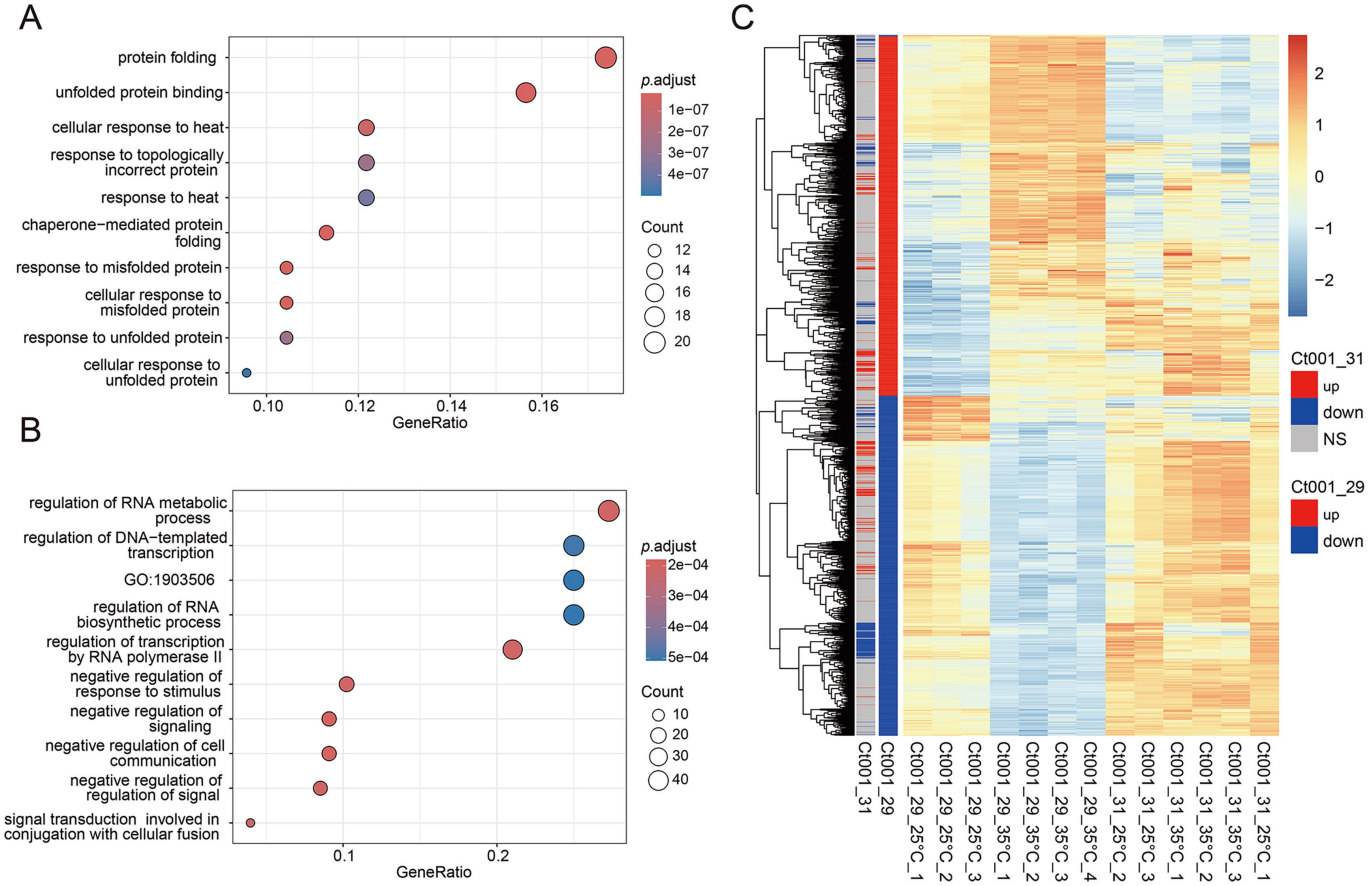
Functional enrichment and expression analysis of differentially expressed genes. **(A)** GO enrichment of up-regulated genes in Ct001_29 cultured at 35 °C. **(B)** GO enrichment of down-regulated genes in Ct001_29 cultured at 35 °C. In panels A and B, “Count” represents the number of genes annotated with a given GO term, “*p*.adjust” represents the adjusted *p*-value from statistical testing, and “GeneRatio” denotes the ratio of input genes annotated in that term. **(C)** Heatmap showing the expression profiles of DEGs across all samples. “up” and “down” represents upregulated and down-regulated genes, respectively, “NS” represents no differential expression.

Furthermore, functional enrichment analysis was performed on the DEGs identified in each haploid. In Ct001_29 cultured at 35 °C, up-regulated genes were significantly enriched in functions related to protein folding and response to heat (Figure 3A), whereas down-regulated genes were enriched in nucleic acid metabolism and signaling regulation (Figure 3B). A total of 22 genes were assigned to the GO terms “protein folding” and “response to heat.” Among them, 14 genes were commonly enriched in both terms, while the other eight were exclusively enriched in “protein folding.” This collection comprises 13 *HSP* genes, together with genes belonging to the AAA + and Pkinase families (Supplementary Table S7). Despite their established roles in heat stress response, heat shock transcription factors (HSFs) were not significantly enriched in our analysis. This is attributable to the modest expression changes of *CtHSFs*, with most exhibiting fold changes below two-fold based on RNA-seq profiles. We subsequently conducted qPCR assays to verify the expression patterns of all five *CtHSFs* annotated in the Ct001_29 and Ct001_31 genomes. The qPCR results were in good agreement with RNA-seq data, demonstrating that the expression levels of most *CtHSFs* varied by less than two-fold (Supplementary Figure S7B). In contrast, no significant functional enrichment was observed among the DEGs in Ct001_31 under the same conditions. Nevertheless, four *CtHSP20*, two *CtDnaJ*, and one *CtHSP90* gene were differentially expressed in Ct001_31 in response to temperature. Across the two haploids, a total of six *CtDnaJ* genes were differentially expressed, one of which was shared between both haploids (hereafter referred to as *CtDnaJ.16*). Transcriptomic data showed that *CtDnaJ.16* was downregulated at 35 °C in Ct001_29 but upregulated at 35 °C in Ct001_31. And qPCR analysis confirmed its significant upregulation in Ct001_31, while no significant change was detected in Ct001_29 (Supplementary Figure S8). No differential expression of *CtHSP70* genes was observed in either haploid. We further analyzed the expression of genes involved in ROS generation and homeostasis. Overall, RNA-Seq data showed higher expression levels in Ct001_31. Both NADPH oxidase genes were robustly expressed in both haploids at 25 °C and 35 °C. Antioxidant genes, however, displayed distinct trends: eight *CtPOD* genes were upregulated in Ct001_31 at 35 °C, one *CtCAT* gene showed elevated expression in Ct001_31 at 25 °C, and *CtSOD* genes exhibited haploid-biased patterns (three favoring Ct001_29, one favoring Ct001_31, and one comparable; Supplementary Figure S9).

Additionally, many genes up-regulated in Ct001_29 at 35 °C were not differentially expressed in Ct001_31. And many genes that were strongly suppressed in Ct001_29 maintained high expression levels in Ct001_31 under both temperatures (Figure 3C). Collectively, these results suggest that the two haploids employ divergent molecular mechanisms and adaptive strategies to support growth under high-temperature conditions. Ct001_29 appears to be temperature-sensitive, whereas Ct001_31 exhibits an innate capacity for thermotolerance.

### 3.4 *CtDnaJ.16* contributes to high-temperature growth adaptation in Ct001_31 but not in Ct001_29

To better understand *DnaJ* family in *C. trogii*, a systematic genome-wide analysis was conducted. A total of 30 *CtDnaJ* genes were identified in each haploid genome, Ct001_29 and Ct001_31. These genes formed strictly allelic orthologous pairs between the two haploids (Supplementary Figure S10) and were designated *Ct29DnaJ.1* through *Ct29DnaJ.30* and *Ct31DnaJ.1* through *Ct31DnaJ.30*, respectively (Supplementary Table S8). Analysis of genetic variation between the allelic pairs revealed 369 SNPs and 15 Indels (Supplementary Table S9). The extent of variation among alleles varied considerably: no sequence differences were detected between *Ct29DnaJ.21* and *Ct31DnaJ.21*, whereas 29 SNPs and one Indel were observed between *Ct29DnaJ.23* and *Ct31DnaJ.23*. Subsequently, seven allelic pairs were randomly selected for experimental validation, which confirmed the accuracy of all identified genetic variants (Supplementary Figure S11).

The gene *CtDnaJ.16* exhibited marked upregulation at 35 °C relative to 25 °C in Ct001_31, whereas no notable difference in expression was observed between the two temperatures in Ct001_29. In the promoter sequences (upstream 2,000 bp) of *Ct29DnaJ.16* and *Ct31DnaJ.16*, there are 11 SNPs and one Indel. These variations may lead to alterations in cis-elements, thereby potentially affecting allele-specific expression. For example, a 9-bp sequence (AGTCAACGC) inserted at position −58 bp in the promoter of *Ct29DnaJ.16* disrupts the binding sites of C2H2 and Zn2C6 (Supplementary Figure S12). Both *Ct29DnaJ.16* and *Ct31DnaJ.16* are predicted to localize to the cytoplasm. Their CDS regions contain three SNPs: c.455 T > A, c.549 C>A, and c.834 C>A, which result in two amino acid substitutions: p. Met152Lys (located within the J domain) and p. Ser278Arg. These amino acid substitutions may affect protein function.

**Figure 4:**
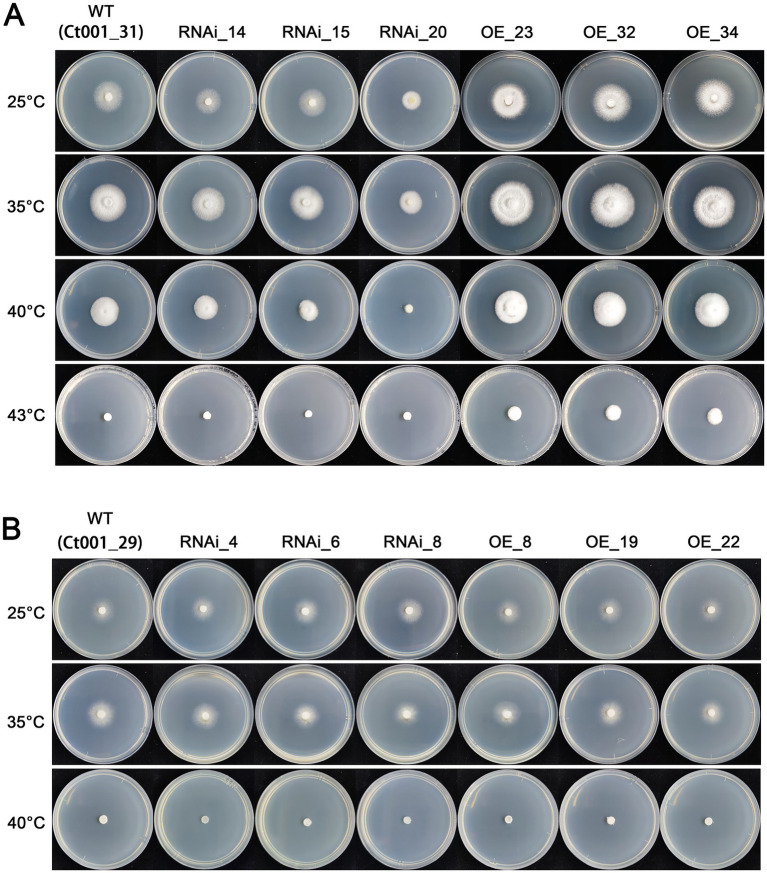
Phenotypes of *CtDnaJ.16* overexpression and RNA interference transformants in two haploid background under different temperature regimes after 2 d of culture. “OE” and “RNAi” represent overexpression and RNA interference transformants, respectively.

Using *A. tumefaciens*-mediated transformation, both OE and RNAi constructs of *CtDnaJ.16* (Supplementary Figure S13) were introduced into each haploid, and three independent transformants per construct were selected for downstream analysis. Following culture at 25 °C and 35 °C, OE transformants of Ct001_29 displayed expression levels 1.57- to 2.23-fold those of WT, while RNAi transformants showed expression ranging from 0.58- to 0.72-fold of WT levels (Supplementary Figure S14). Similarly, in the Ct001_31 background, OE transformants exhibited 1.59- to 2.62-fold expression those of WT, while RNAi transformants showed levels between 0.47- and 0.90-fold of WT expression (Supplementary Figure S14). To evaluate the role of *CtDnaJ.16* in thermotolerance, transformants and WT were cultured across a range of temperatures. In the Ct001_31 background, OE transformants exhibited enhanced heat tolerance, with germination observed even at 43 °C, whereas RNAi transformants showed reduced thermotolerance, most notably the RNAi20 transformant, which failed to germinate at 40 °C (Figure 4A). In contrast, in Ct001_29, neither OE nor RNAi transformants differed significantly from WT in their heat response (Figure 4B). The *in situ* DAB and NBT staining signals did not clearly distinguish the physiological differences between the transformants and the WT (Supplementary Figure S15). We further selected representative transformants for qPCR analysis to measure the transcript levels of genes involved in protein folding, heat response, and ROS production pathways (Supplementary Figure S16). The qPCR results revealed that, in the Ct001_31 haploid, silencing of *CtDnaJ.16* at 35 °C significantly upregulated the expression of *CtClpB* (Tt31c01g0010311), four tandemly arranged *CtHSP20* genes, *CtHSP90* (Tt31c28g0118361), and the gene encoding the HSP90 ATPase activator, suggesting that these genes may compensate for the functional defects caused by *CtDnaJ.16* depletion. In contrast, in the Ct001_29 haploid, *CtDnaJ.16* silencing at 35 °C significantly induced only the four tandem *CtHSP20* genes. For the two NADPH oxidase genes involved in ROS production (Tt31c10g0078771 and Tt31c01g0009511), silencing *CtDnaJ.16* at 25 °C markedly activated their transcription in both genetic backgrounds. However, upon temperature elevation to 35 °C, the inductive effect of *CtDnaJ.16* silencing on Tt31c10g0078771 was substantially attenuated. These results clearly demonstrate that *CtDnaJ.16* plays divergent biological roles in the two haploid backgrounds.

Given that DnaJ proteins typically function as co-chaperones through interactions with HSP70s, we tested whether the thermotolerance role of Ct31DnaJ.16 involves direct partnership with CtHSP70s. Y2H assays were performed against all seven CtHSP70s encoded in the Ct001_31 genome; however, no detectable interactions were observed, even following high-temperature treatment (Supplementary Figure S1). Together with the RNA-Seq data showing that *CtHSP70* family members were not differentially expressed across different temperatures in Ct001_31, these findings indicate that the heat-protective function of CtDnaJ.16 in this haploid is unlikely to rely on canonical binding to CtHSP70s.

## 4 Discussion

The monokaryotic stage in macrofungi, typically derived from germinated spores or protoplast regeneration, provides a genetically homogenous system ideal for genomic studies and breeding programs (Wang et al., 2021; Chen et al., 2022; Pan et al., 2024; Wang et al., 2024a; Wang et al., 2024b; Zhou Y. Y. et al., 2024). In contrast, phenotypic traits in dikaryotic strains result from the combined activity of two genetically distinct nuclei, complicating the dissection of underlying molecular mechanisms. In this study, two sexually compatible haploids, Ct001_29 and Ct001_31, derived from the same parental strain, were employed. These haploids exhibited marked differences in physiological responses and thermotolerance when cultured at 25 °C and 35 °C, demonstrating clear haploid-specific adaptations to identical thermal stress. Investigating the differential molecular mechanisms within clear genetic background offers a novel and powerful approach to elucidate the basis of heat tolerance in macrofungi.

Accurate genome annotation serves as the foundational for functional genomics studies. A recent investigation into fungal transcription factors containing only the middle homology domain revealed that over 90% lacked annotated DNA-binding domains, a direct consequence of genome annotation errors (Mayer et al., 2023). Although high-quality genome assemblies have become increasingly accessible, achieving precise annotation remains a persistent bottleneck, often necessitating iterative refinement (Cheng et al., 2017; Li et al., 2019). We previously established a gold-standard annotation for *Ganoderma lingzhi* through manual curation, revealing exceptional genomic features such as non-canonical splicing sites and abundant alternative splicing (Wang et al., 2024a; Wang et al., 2024b). Building on this methodological framework, a comprehensive manual upgrade of the Ct001_31 genome annotation was undertaken in the present work. Among the 12,953 high-confidence genes ultimately obtained, only 45.20% remained unmodified, while the remaining 54.80% underwent structural modifications, underscoring the critical importance of manual curation. This refined, high-confidence annotation subsequently served as an essential reference for RNA-Seq analyses in this study.

RNA-Seq analyses of the two haploids revealed strikingly divergent gene expression strategies in response to high-temperature, demonstrating that the two haploids employ distinct molecular mechanisms and adaptation strategies for growth under high temperature. From an evolutionary perspective, such intrapopulation polymorphism in stress-response strategies likely represents a significant adaptive advantage. Rather than relying on a single response mode when facing environmental fluctuations, the population retains multiple potential molecular pathways for adaptation, thereby enhancing its overall adaptive capacity and resilience (Wang et al., 2022). This study provides direct transcriptomic evidence supporting the adaptive significance of maintaining genetic and expression diversity in natural populations of macrofungi. In strains Ct001_29 and Ct001_31, *CtHSFs* did not undergo significant expression alteration. This pattern is in line with our previous study (Wang et al., 2024a; Wang et al., 2024b). Specifically, when the dikaryotic strain Ct001 was cultured at 25 °C and 35 °C, or subjected to a temperature shift from 25 °C to 35 °C, the expression fold changes of all five *CtHSF* genes were below two-fold. It is likely that the activity of HSF proteins under high temperature is mainly regulated by post-translational modifications (e.g., phosphorylation; Sorger and Pelham, 1988). In addition, transcription factors of Zn2C6 and C2H2 families also play key roles in regulating thermotolerance of *C. trogii* (Wang et al., 2024a; Wang et al., 2024b).

A total of 30 allelic *CtDnaJ* genes were identified in *C. trogii*, a repertoire comparable to that of *L. edodes* (Wang et al., 2019). The distribution of genetic variation among these alleles was notably uneven, exhibiting a pattern of conservation interspersed with divergence. This co-occurrence of conserved and variable sequences may reflect the action of balancing selection (Charlesworth, 2006), a mechanism that maintains genetic polymorphism within the population over the long term. By preserving essential biological functions while providing raw material for natural selection, this genetic architecture enables adaptation to changing environmental conditions. Whether these sequence differences translate into meaningful functional variation, including alterations of protein activity, subcellular localization, or interaction networks, awaits further experimental validation.

Functional validation via *Agrobacterium*-mediated transformation demonstrated that *CtDnaJ.16* was essential for thermotolerance in Ct001_31 but may functionally redundant in Ct001_29, underscoring its haploid-specific role and functional divergence between alleles. This finding highlights the importance of conducting haplotype-level functional genomics studies in non-model organisms for understanding adaptive evolution. Moreover, the background-dependent role of *CtDnaJ.16* may involve unique interactors or regulatory pathways specific to each haploid genetic context, a premise that warrants future investigation.

Given the canonical role of DnaJ proteins as co-chaperones for HSP70s, Y2H assays were employed to test for direct interaction between CtDnaJ.16 and all seven CtHSP70s encoded in the Ct001_31 genome. Surprisingly, no interaction was detected. This finding aligns with a report in *Magnaporthe oryzae*, in which two HSP40s (MHF16 and MHF21) also showed no direct Y2H interaction with multiple HSP70s (Yi and Lee, 2008). Several non-mutually exclusive explanations may account for this result: CtDnaJ.16 may function independently of CtHSP70s; the interaction might be transient or too weak to be captured by Y2H; or it may require an intermediary protein to facilitate binding. Classical studies investigating HSP40-HSP70 partnerships have often relied on biochemical methods, such as ATPase activity assays (Kotler and Street, 2023) and pull-down assays (Qian et al., 2002), rather than Y2H alone. Therefore, future work should employ such biochemical methods to definitively characterize the potential partnership between CtDnaJ.16 and CtHSP70s, and to explore alternative interactors that likely mediate its haploid-specific function in thermotolerance.

## 5 Conclusion

Utilizing manually curated and upgraded genome annotations, this study employed comparative transcriptomics to investigate the molecular mechanisms underlying high-temperature adaptation in two haploids with differing thermotolerance. The two haploids exhibited distinct transcriptional response mechanisms under high-temperature condition. Further analysis revealed that *CtDnaJ.16* is involved in growth adaptation to high-temperature in the Ct001_31 background, but not in Ct001_29, indicating functional divergence of this gene across different haploid contents. These findings suggest that allelic genes may undergo functional divergence in different genetic backgrounds, which holds significant biological implications for understanding the interaction between environmental adaptation and genetic background in species.

## Data Availability

The datasets presented in this study can be found in GPGD (Liao et al., 2021): http://www.gpgenome.com/species/62343.

## Footnotes

1
https://ccb.jhu.edu/software/stringtie/gffcompare.shtml

2
https://sparql.uniprot.org/

3
https://cran.rproject.org/web/packages/pheatmap/

4
https://services.healthtech.dtu.dk/services/DeepLoc-2.1/

5
https://www.htslib.org

6
https://jaspar.elixir.no/
