# Supplementary material for: From phenotype to mechanism: background-dependent function of CtDnaJ.16 in thermotolerance-divergent Coriolopsis trogii haploids
Source: Front Microbiol. 2026 Jul 10;17:1852348. doi: 10.3389/fmicb.2026.1852348 (PMC13395996; doi:10.3389/fmicb.2026.1852348)
Supplement: Supplementary file 1 [file Data_sheet_1.pdf]

Supplementary Material

This Supplementary Material contains 16 figures and 9 tables.

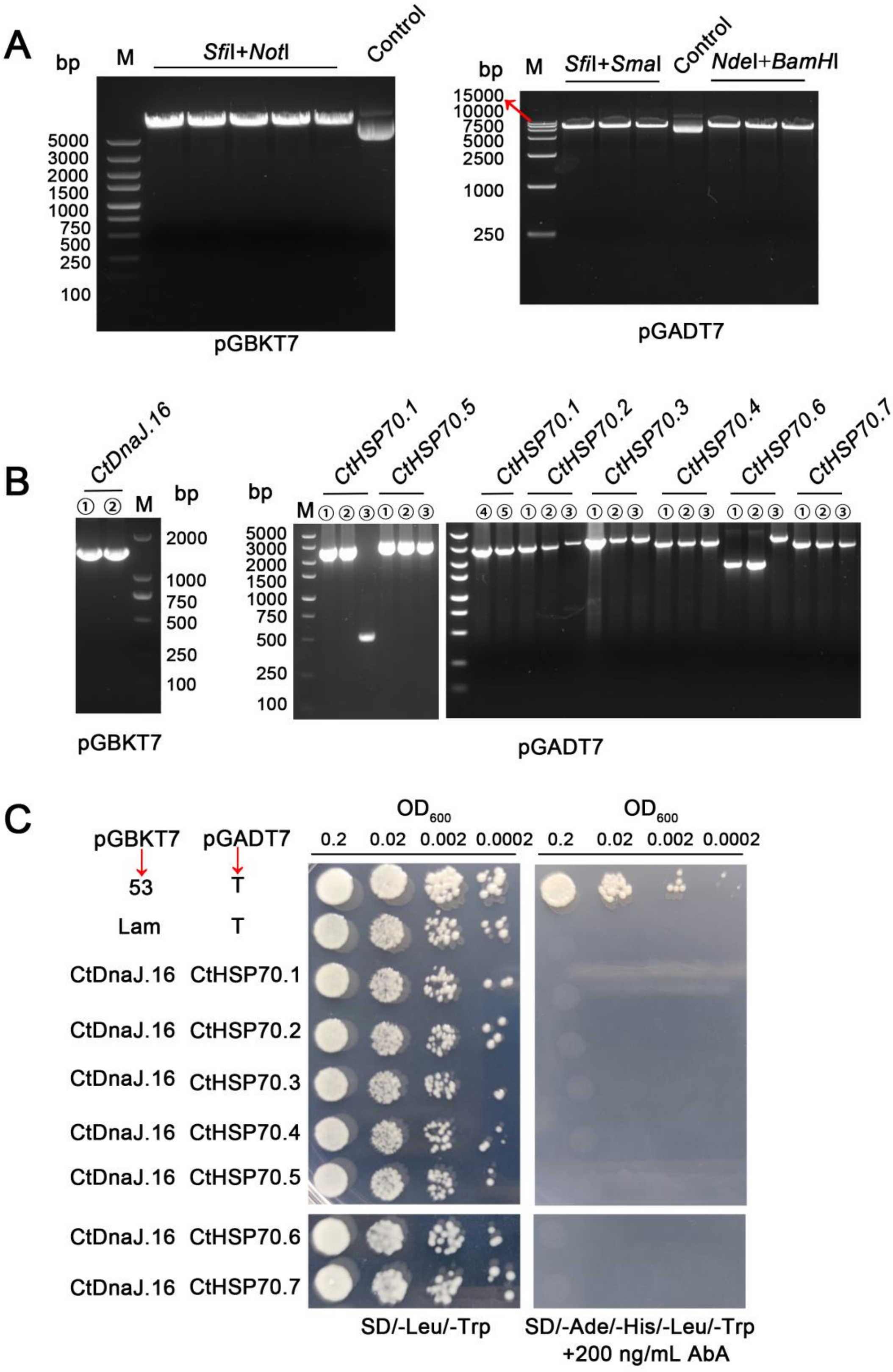

Supplementary Figure S1 Verification of protein-protein interactions using the yeast two-hybrid system. (A) Validation of plasmid constructs by double enzyme digestion. (B) Confirmation of recombinant vectors via PCR and Sanger sequencing. (C) Screening of protein-protein interactions on selective culture plates.

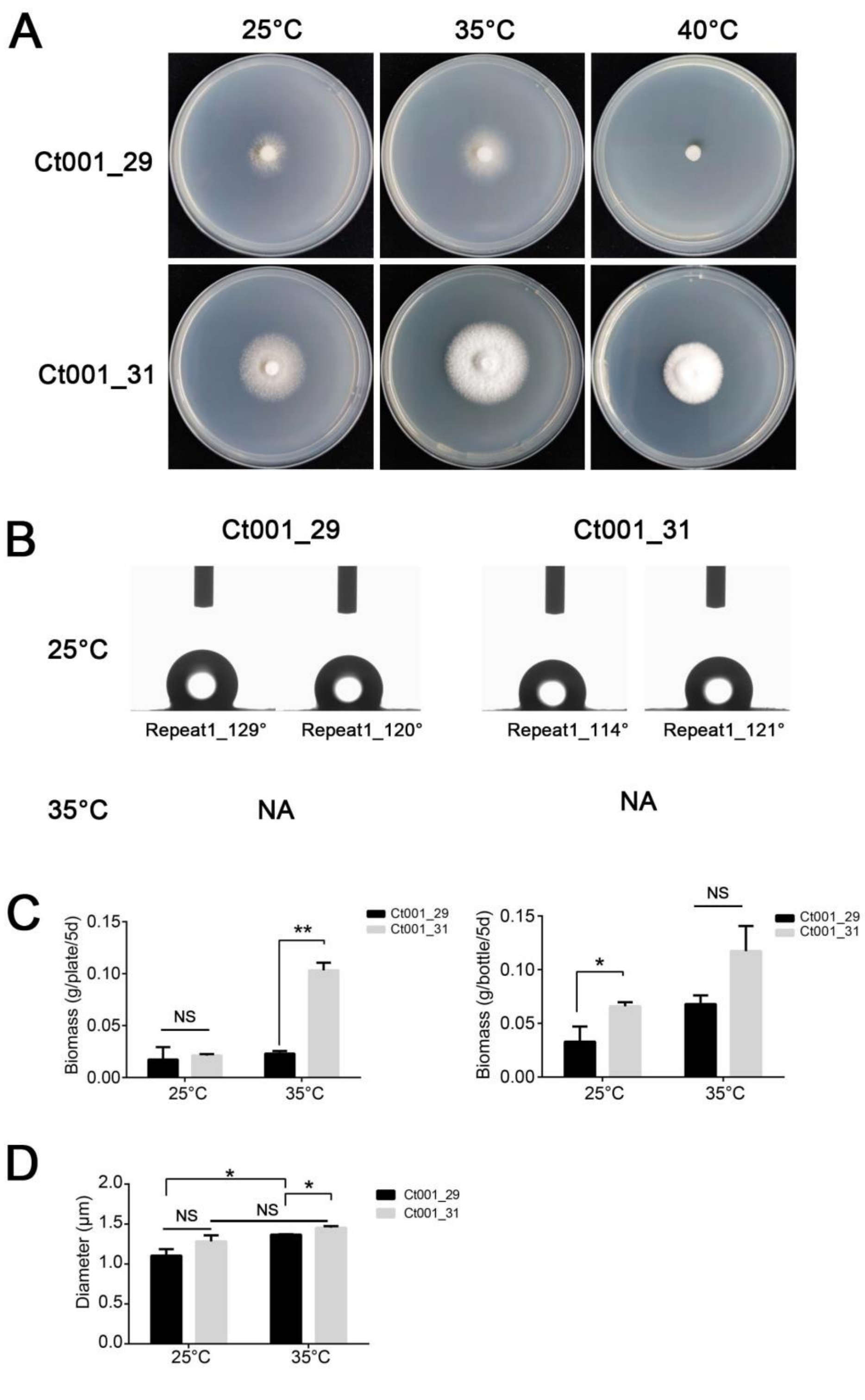

Supplementary Figure S2 Analysis of biological characteristics of Ct001_29 and Ct001_31 at different temperatures. (A) Mycelium cultured on PDA plates for 48 h. (B) Hydrophobicity of mycelia, "NA" represents data not collected under the corresponding condition. (C) Biomass. (D) Quantification of hyphal diameters using scanning electron microscopy. "*", $p < 0.05$; "**", $p < 0.01$, "NS", not significant.

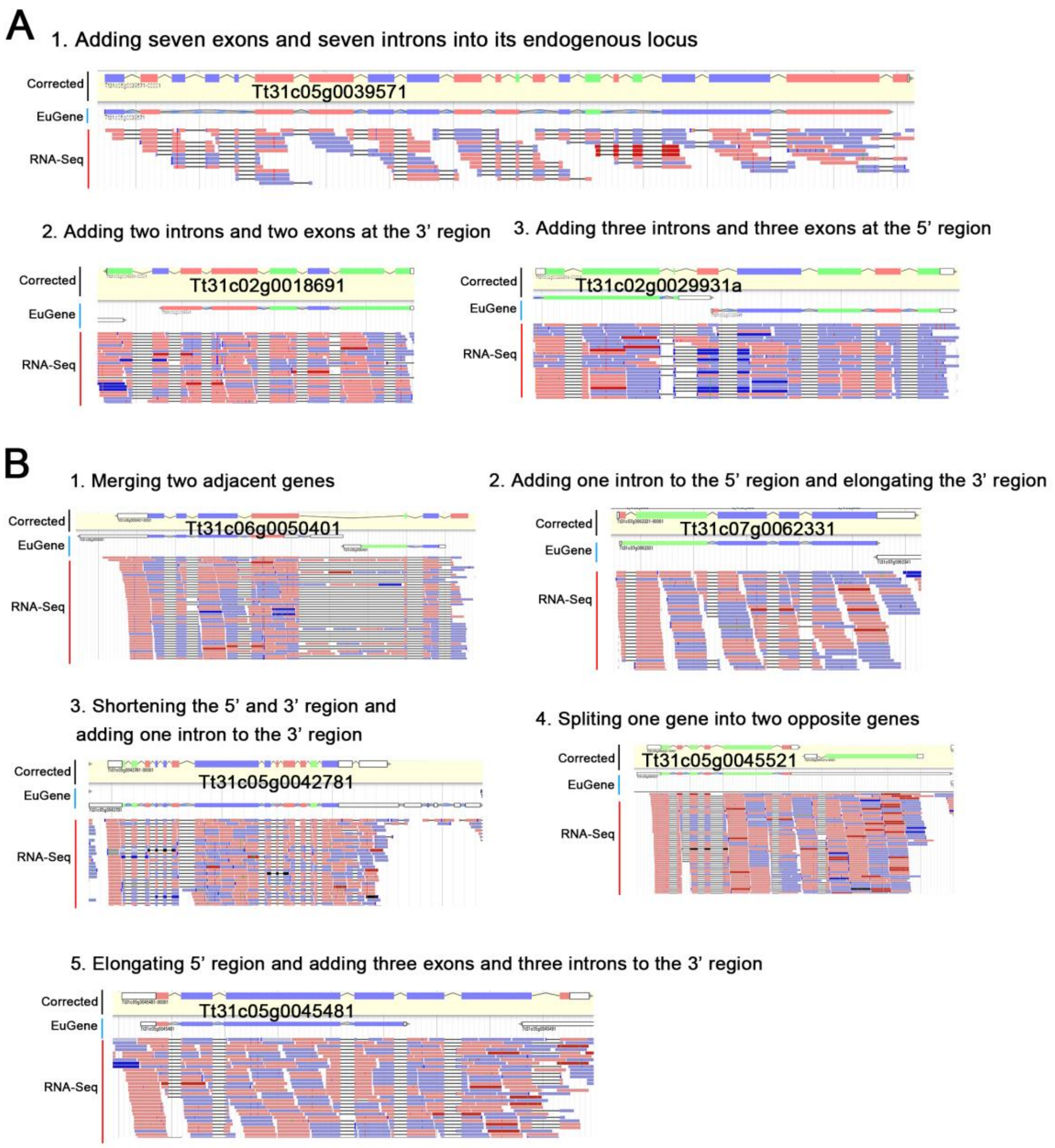

Supplementary Figure S3 Manual correction of genes in genome of Ct001_31. (A) *GH* genes. (B) *DnaJ* genes.

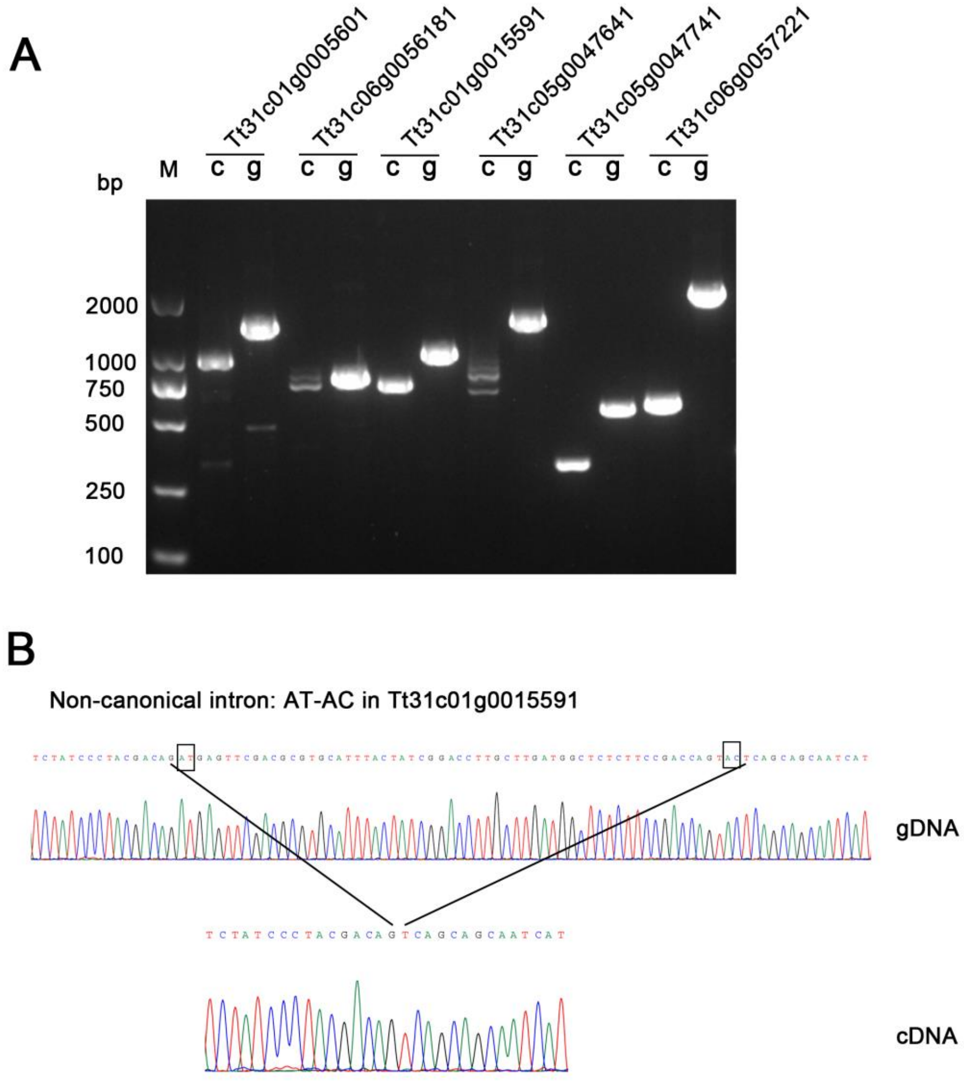

Supplementary Figure S4 Validation of non-canonical introns. (A) Agarose gel electrophoresis. (B) Sanger sequencing chromatogram. “c” and “g” represent cDNA and genomic DNA, respectively.

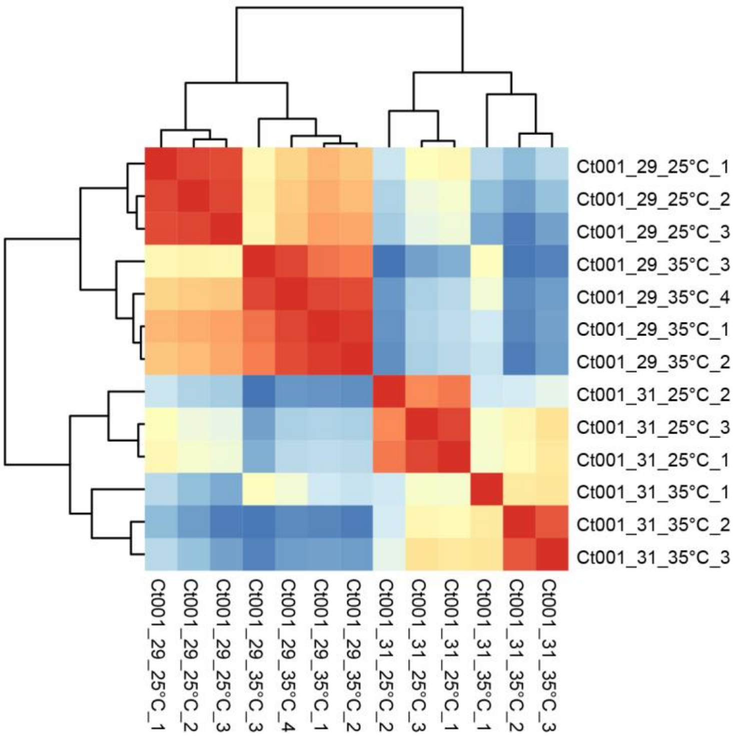

Supplementary Figure S5 Cluster analysis of RNA-Seq data from Ct001_29 and Ct001_31 mycelia cultured at 25°C and 35°C.

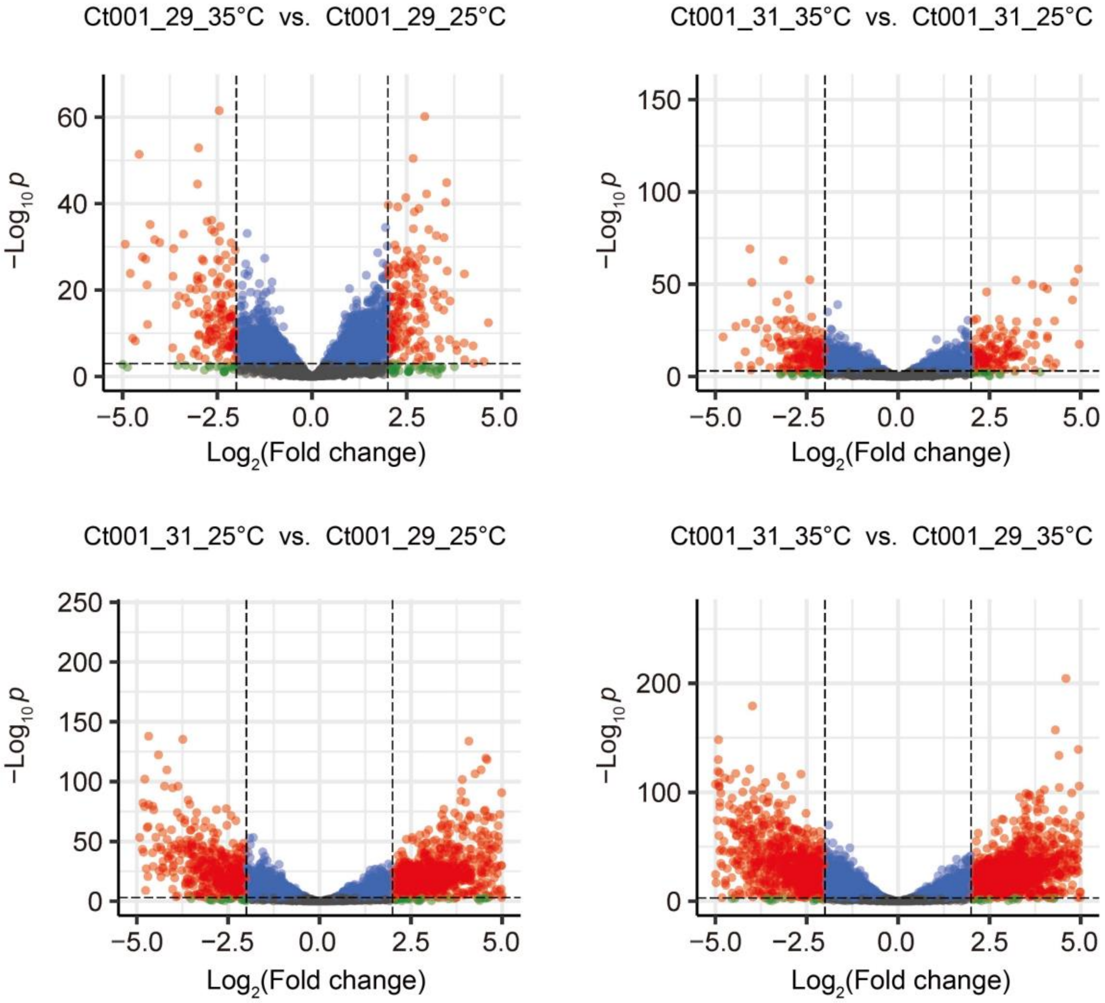

Supplementary Figure S6 Volcano plots of differential gene expression analysis.

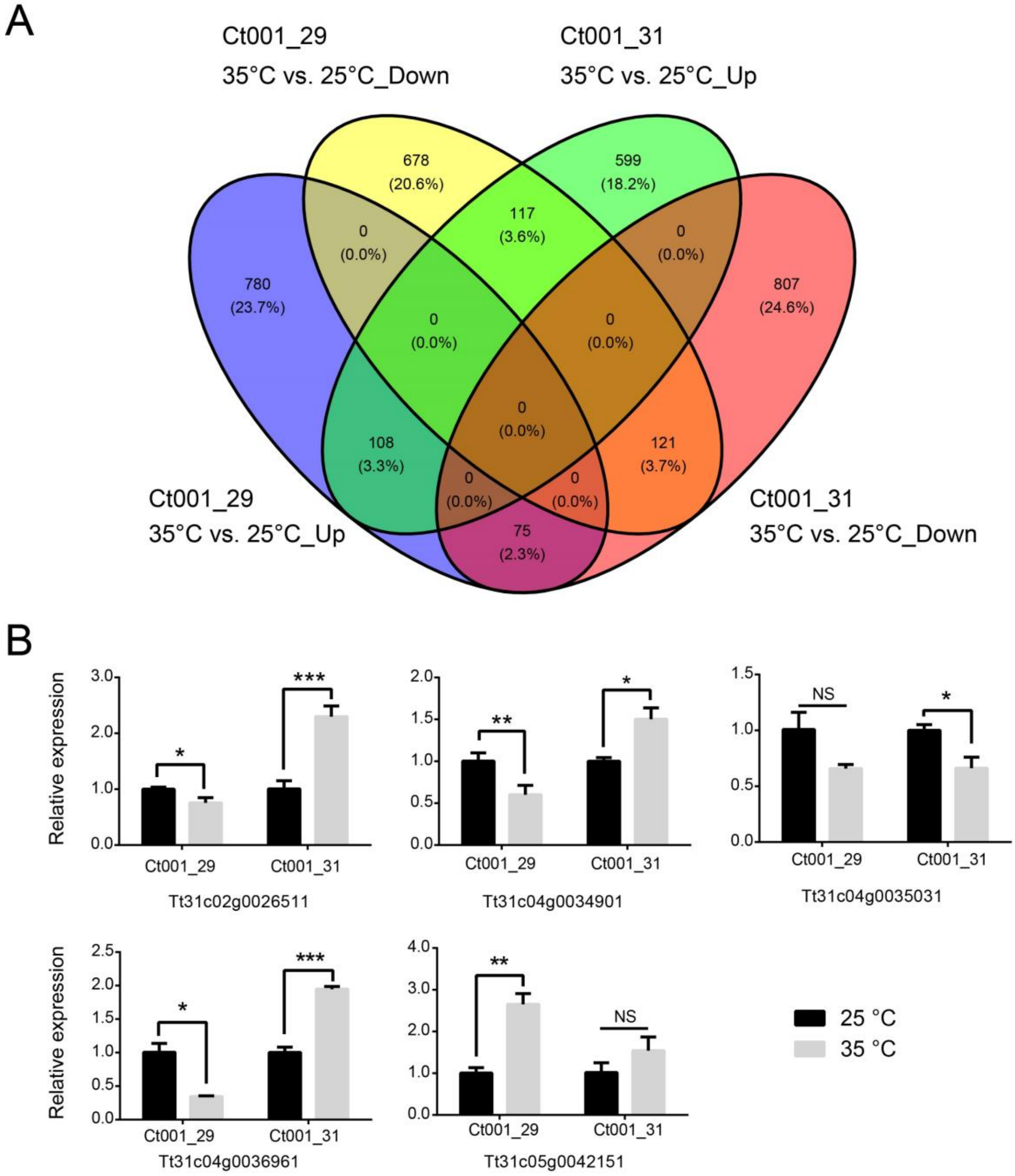

Supplementary Figure S7 Venn diagram of differentially expressed genes and qPCR results of *CtHSFs*.

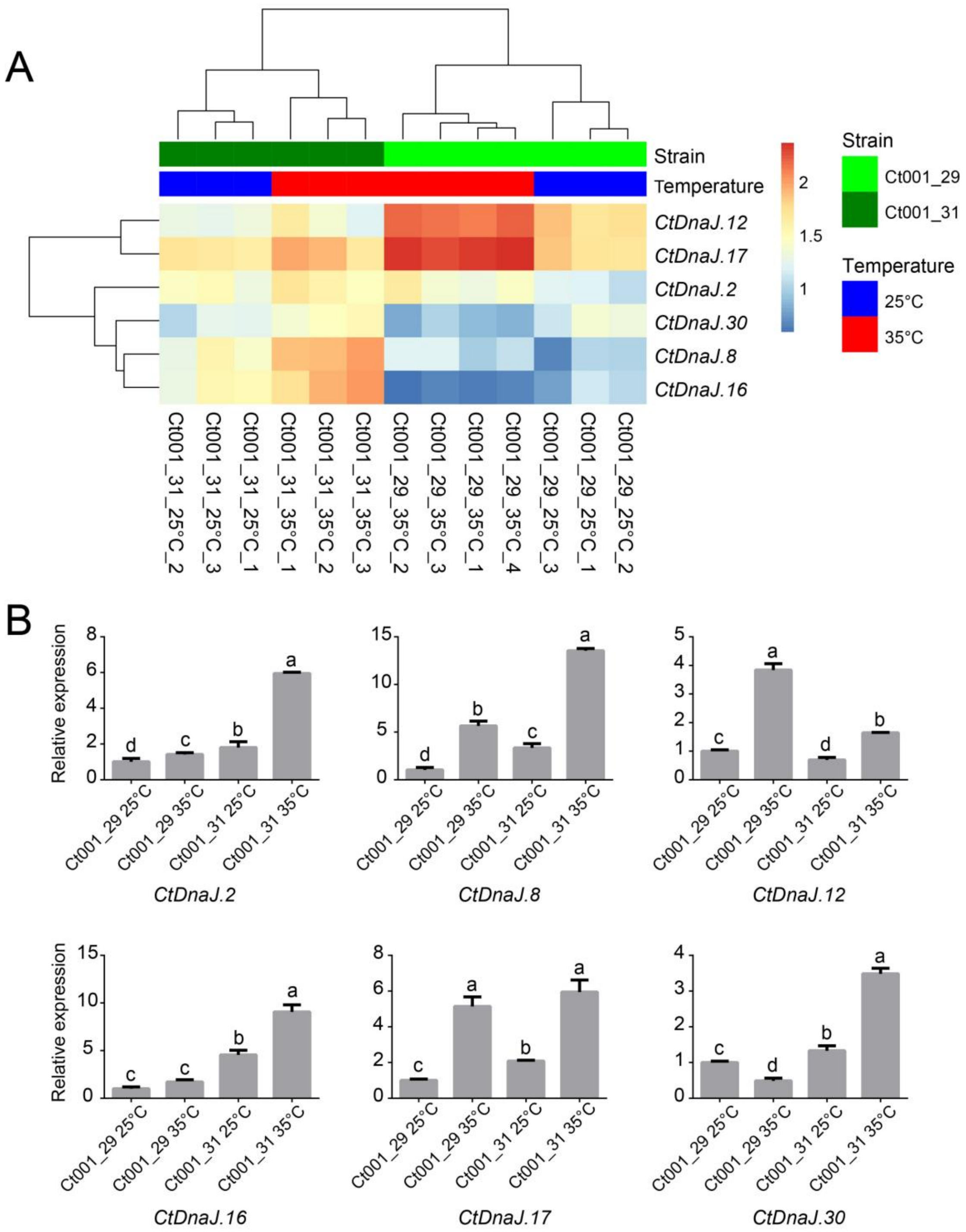

Supplementary Figure S8 Differentially expressed *CtDnaJ* genes. (A) Heatmap. (B) qPCR results. Different lowercase letters indicate significant differences, $p < 0.05$.

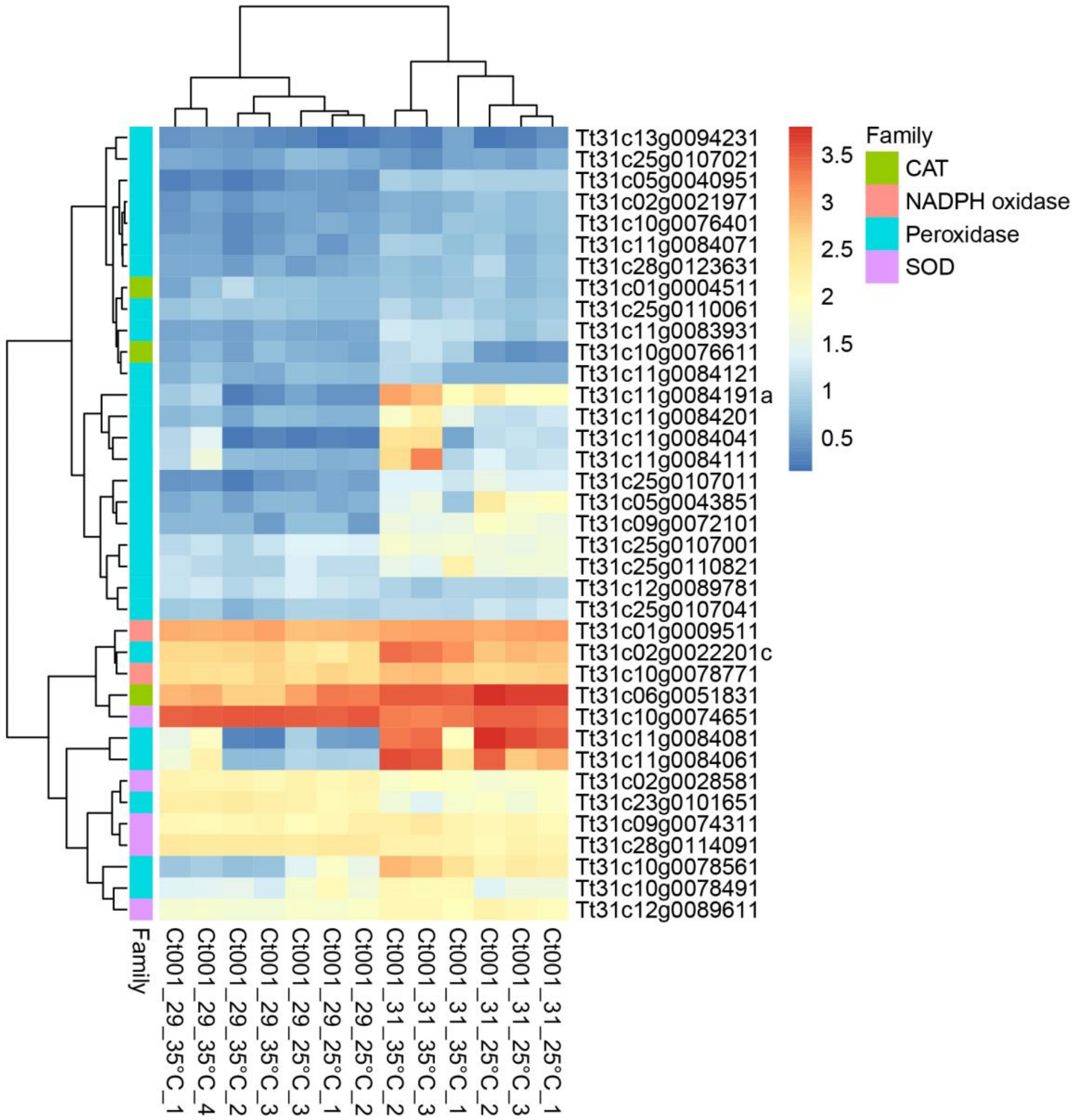

Supplementary Figure S9 Expression of genes involved in ROS generation and ROS homeostasis.

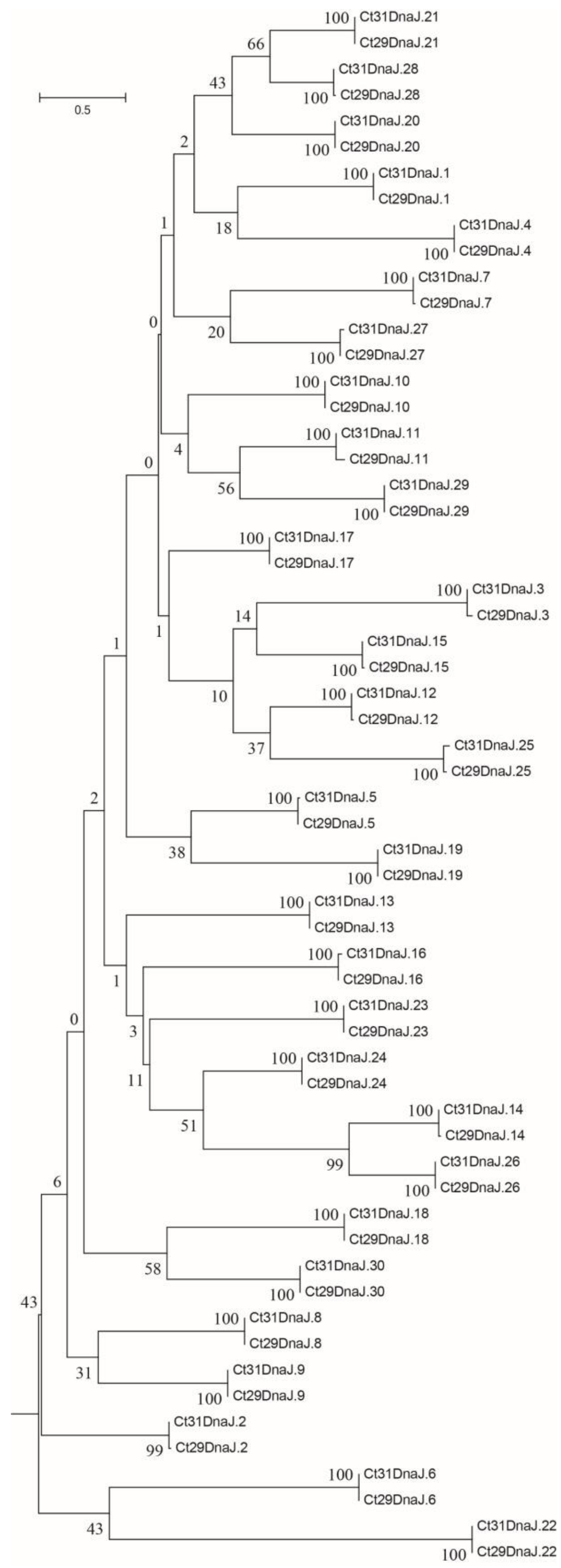

Supplementary Figure S10 Maximum Likelihood tree of allelic *CtDnaJ* genes. The tree was constructed using full-length protein sequences with a bootstrap value of 100.

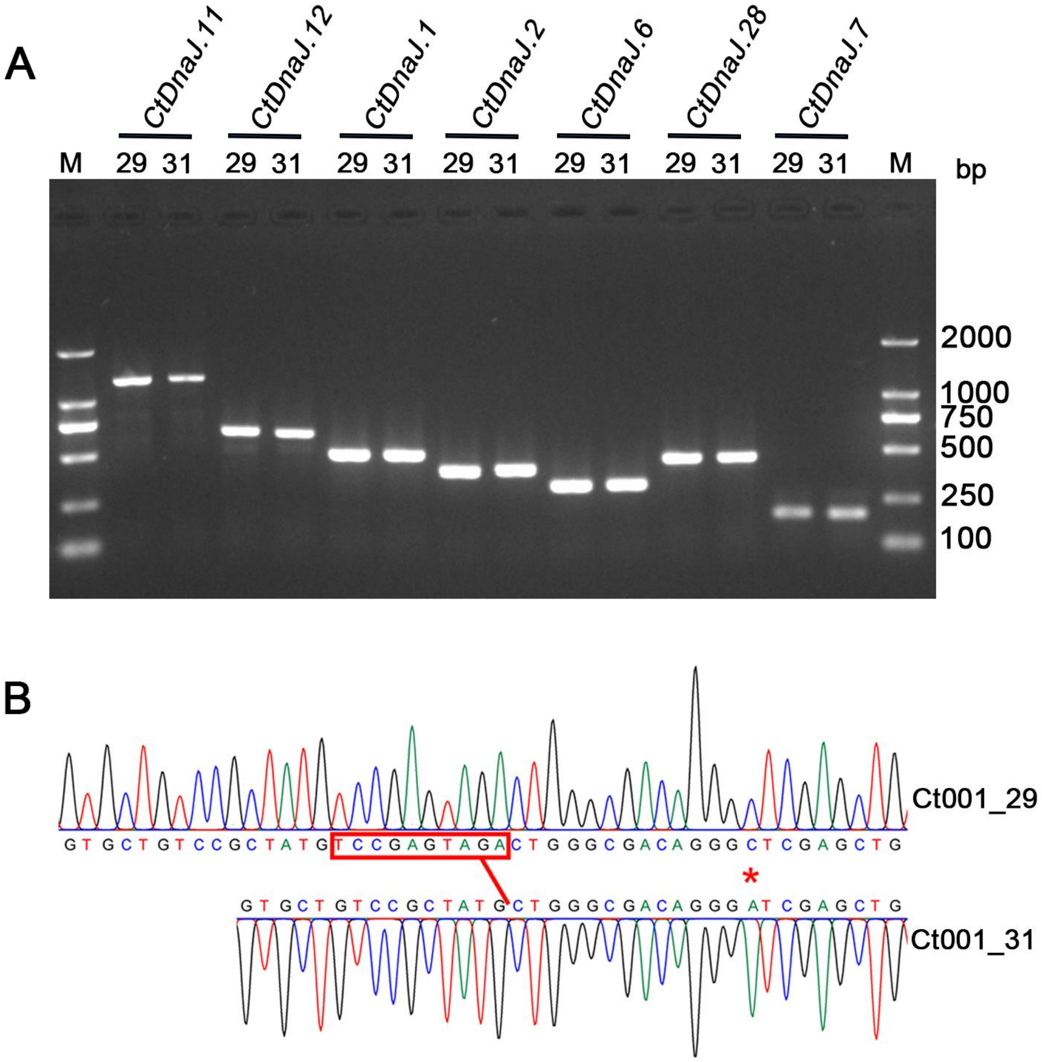

Supplementary Figure S11 Validation of genetic variations between *CtDnaJ* alleles. (A) Agarose gel electrophoresis. (B) Sanger sequencing chromatogram. “29” and “31” represent Ct001_29 and Ct001_31, respectively.

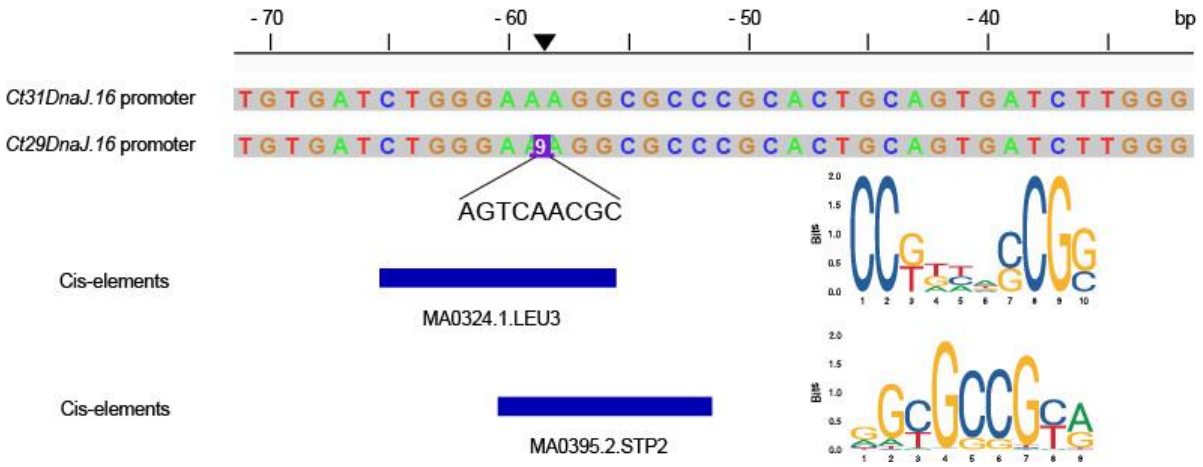

Supplementary Figure S12 A 9-bp insertion disrupts C2H2 and Zn2C6 binding sites in the promoter of *CtDnaJ.16*.

A

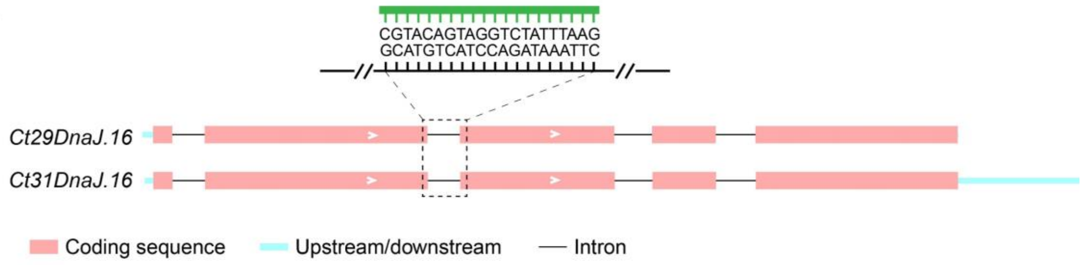

B

GCATGTCATCCAGATAAATTCTCGAGAATTTATCTGGATGACATGTTTTTT

Supplementary Figure S13 Construction of RNA interference vector of *CtDnaJ.16*. (A) Schematic diagram of RNA interference of *Ct29DnaJ.16* and *Ct31DnaJ.16*. Arrows indicate the direction of transcription. (B) Sequences of the hairpin RNA constructs.

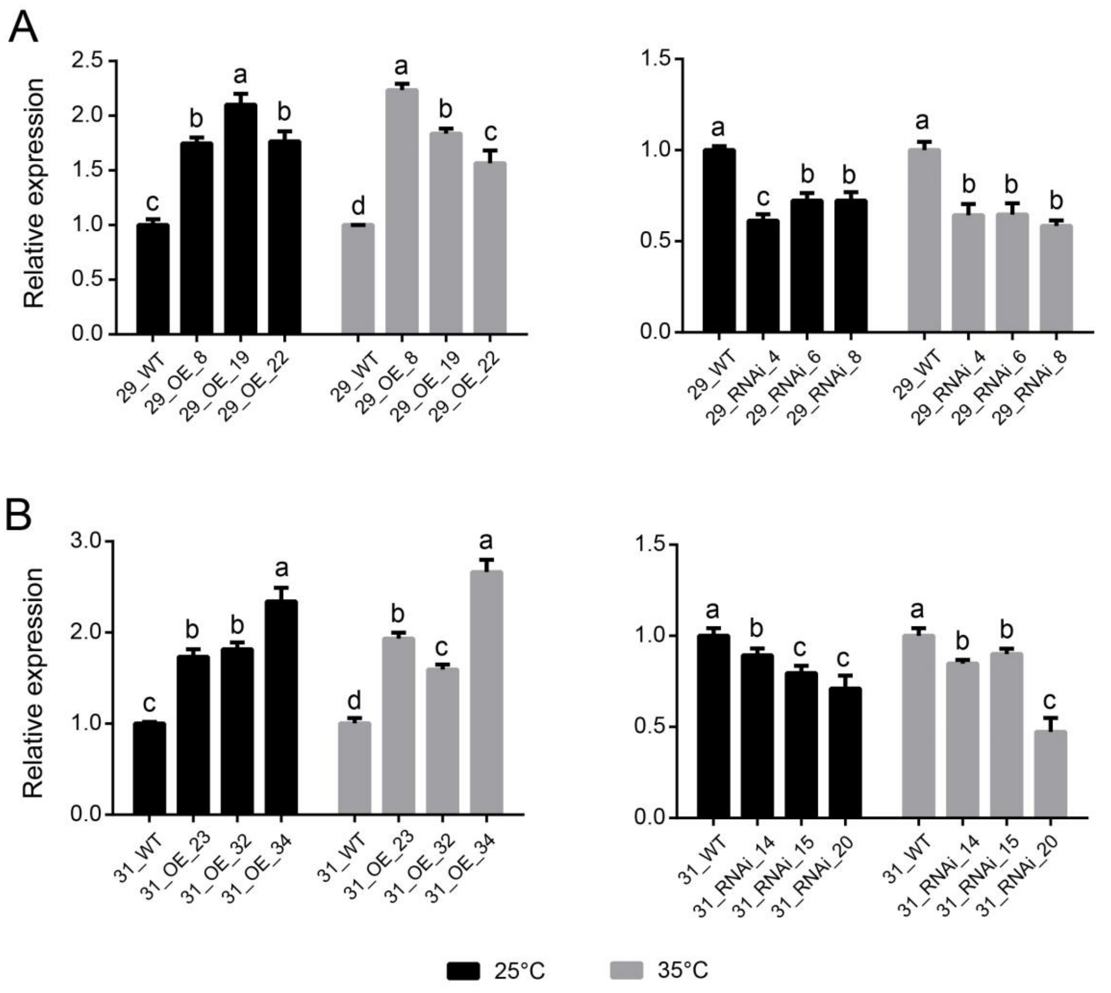

Supplementary Figure S14 Expression validation of *CtDnaJ.16* in transformants. (A) Relative expression levels of *CtDnaJ.16* in Ct001_29 transformants. (B) Relative expression levels of *CtDnaJ.16* in Ct001_31 transformants. “29” and “31” represent Ct001_29 and Ct001_31, respectively. Different lowercase letters indicate significant differences, p < 0.05.

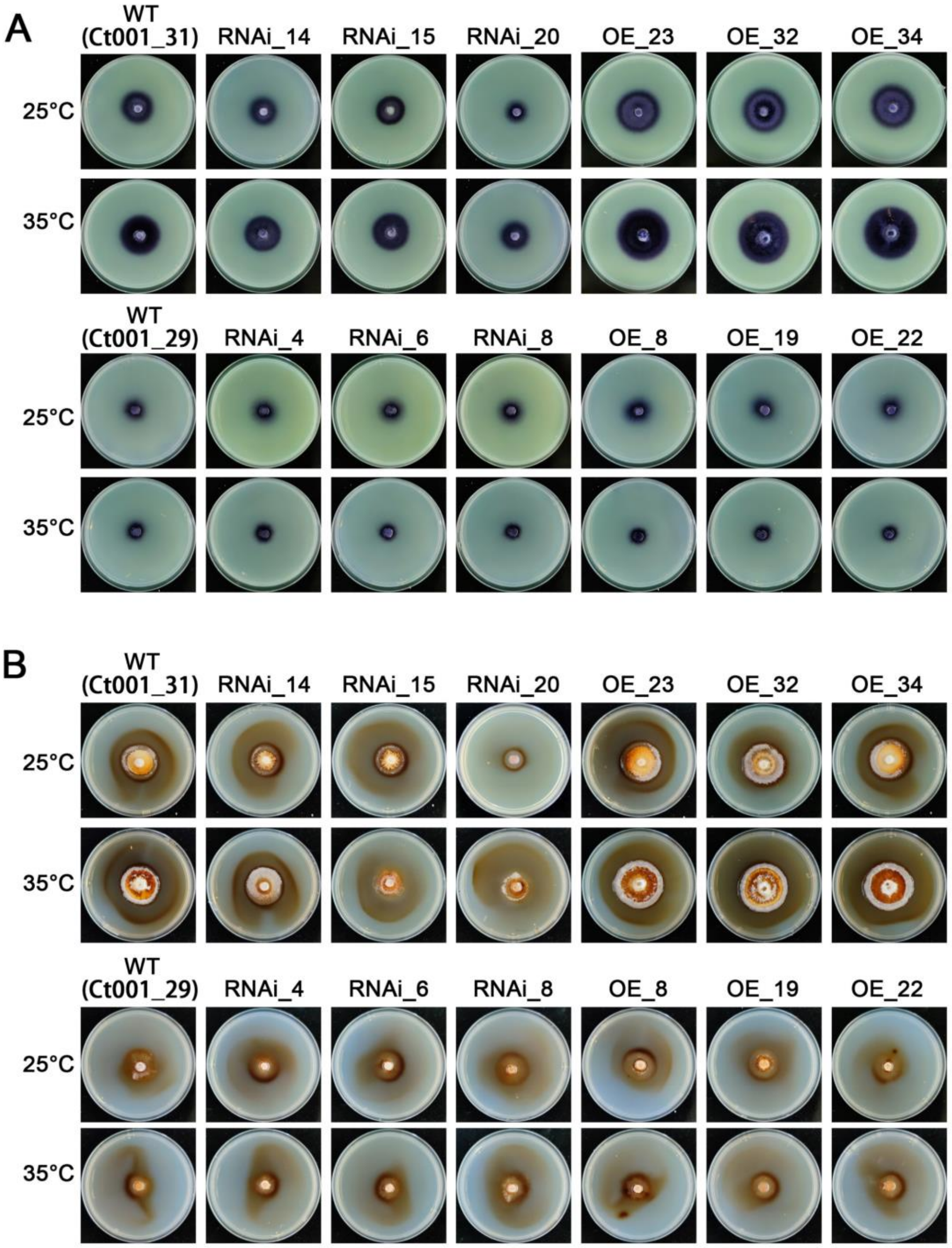

Supplementary Figure S15 In situ NBT (A) and DAB (B) staining of *CtDnaJ.16* transformants.

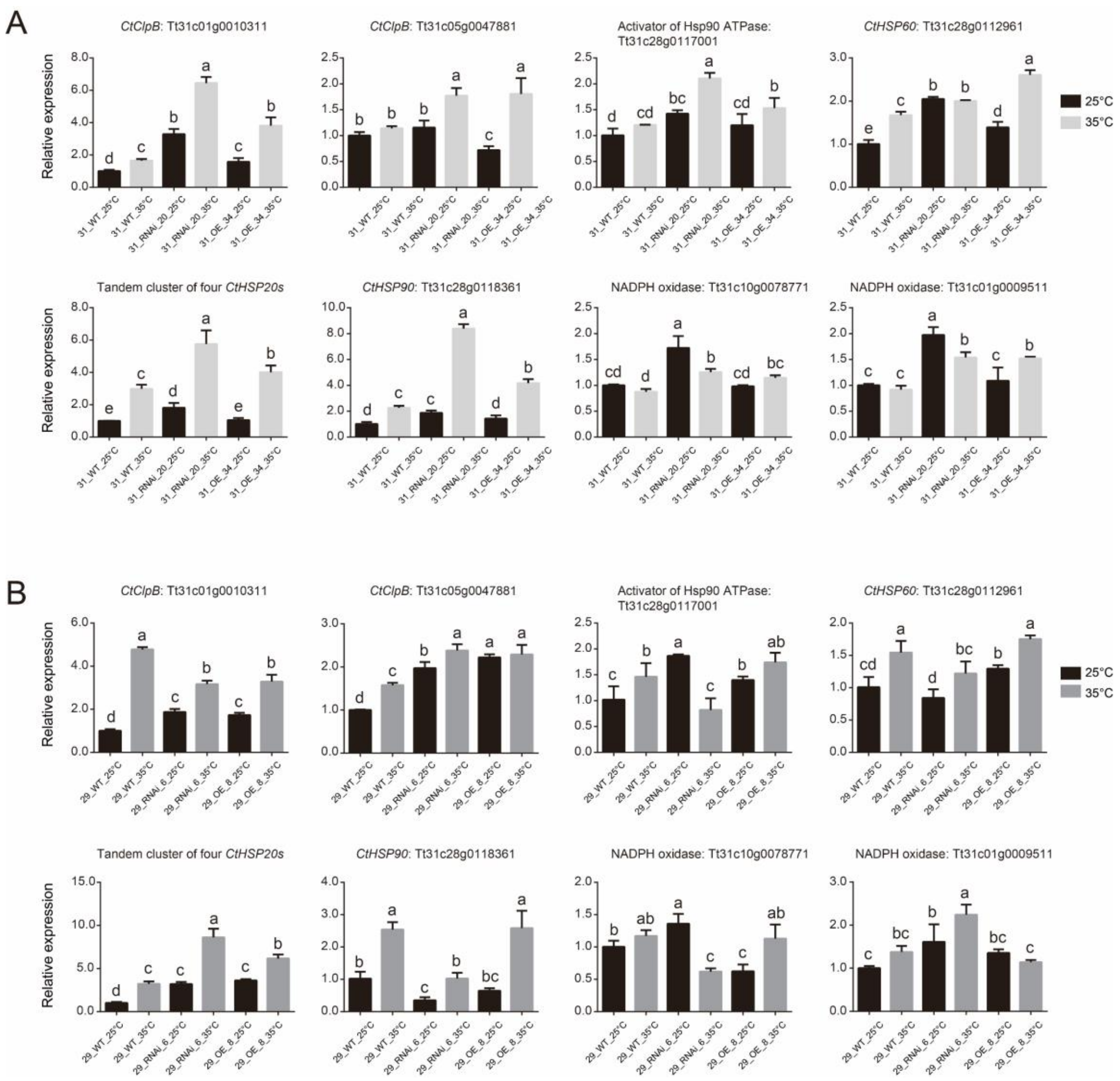

Supplementary Figure S16 Detection of transcript levels of genes involved in protein folding, heat response, and ROS production in *CtDnaJ.16* transformants. “29” and “31” represent Ct001_29 and Ct001_31, respectively. Different lowercase letters indicate significant differences, $p < 0.05$.

Supplementary Table S1 Primers for qPCR analysis

Genes	Forward primer (5'-3')	Reverse primer (5'-3')	Length (bp)	Note
CtDnaJ.2	TCCTCGGCGTGCAGCAGAAT	AACCGCTCCGTGGCTGTCTT	130	-
CtDnaJ.8	TCTCCTCGGCGGGTCAATGTT	GAGCGTCGTGGTCCTTCAATGG	273	-
CtDnaJ.12	TCATGCTGCGGTTCTGCTTGC	CGAGGTCTCGAAGGTCGTCCAT	251	-
CtDnaJ.16	CGACGGCGACTTGGAAATGGTT	AAGTAGGAGAGCTGGCGGAAGG	216	-
CtDnaJ.17	GCGACAAGCAGAAGCGAACCA	CTCCACCGAAACCTCCACCCAT	285	-
CtDnaJ.30	TCGCTGGAGGTGGTCAAGT	GTAGTMGTCGTCGTCATAGTCA	240	-
Tt31c10g0078771	ACTCGCACCACCTCTTCATC	TGACCGTCTTCTCCTTCTTGAT	261	NADPH oxidase
Tt31c01g0009511	ACTTCCTCCGCATCAACATCT	GCCTGACTCATCCTTCCGTAA	169	NADPH oxidase
Tt31c01g0010311	CACCACCACCAGATGAGA	CTTGAGCGATGTAGGAGTC	102	ClpB (AAA+ family)
Tt31c05g0047881	GCGAGTGGTTGATGAAGGAG	TAGACGATGCGGCGATAGAA	216	ClpB (AAA+ family)
Tt31c28g0112961	CCATCTACTCGGAAGGTGTCAA	GCGGTGGTGGTGATTGTCT	133	HSP60
Tt31c28g0118361	GGCTTCTACTCCGCATACCT	GACCTCCTTCTCAACCTCCTT	285	HSP90
Tt31c28g0117001	ATGCGGTGCTGAAGAAGAAG	GCGTCCACATCGGTATCCT	165	Activator of Hsp90 ATPase
CtHSP20	TCTTCACCGAGCCGTTCTAC	CCGTTACGAGGTTCTTGTCTTG	180	Four tandem *CtHSP20s*
Tt31c04g0036961	GCTTCCTTCGTCCGTCAAC	TCAAGTGCGTCTCGTCTGT	131	*CtHSF*
Tt31c04g0035031	AGACTGATGATTGGCGATGGTA	GGCGTAGCGTTCCGATGAA	126	*CtHSF*
Tt31c04g0034901	AGCCATATCCGCAGACCTATC	TCGCCGTGTARTTGTTCTCT	180	*CtHSF*
Tt31c02g0026511	CCGAGGTGGTAGCCAACTT	GGACAGTGAGAGGAGGAAGG	216	*CtHSF*
Tt31c05g0042151	YTAGAAGGCGGCGAGAGTC	GCTGATGGAGTGTAACGAGTG	129	*CtHSF*
Tt31c28g0121441	CGAGTGAACTTATGAGCCTAATGA	CGAACTTAATGGCAGGAGGTG	192	Reference
GAPDH	CCGTTGGTGCTGATTACA	CGTTGGAGATGACTTGGTA	183	Reference

Note: “-” represents none.

Supplementary Table S2 Primers for validating the gene structures of *DnaJ* and glycoside hydrolase (*GH*) genes, as well as non-canonical introns

Genes	Forward primer (5'-3')	Reverse primer (5'-3')	cDNA (bp)	gDNA (bp)	Amplification products
Tt31c05g0045481	CGAGGTTCTTGGAATAGACA	CCAGATATTGGCGTTCAGT	1,500	-	*DnaJ*
Tt31c06g0050401	CTGTAGAGTCAACACCAGAA	TGAGAACTTAGACCGAAGAG	1,012	-	*DnaJ*
Tt31c06g0053901	TCTGGTGCTTGAGAATCG	TGATCGCAGAAGGTTGAC	1,939	-	*DnaJ*
Tt31c07g0062331	ATGTTCTCCTATGTCCTCGGCTC	CTACTTCTTCACGAGGCCTTCC	999	-	*DnaJ*
Tt31c05g0045521	GCCCTCTTCACATCTATCC	GATGCCTGACTGTCCTTC	670	-	*DnaJ*
Tt31c05g0042781	CGACTCCGTAATCAAGATTG	CGATGATGGATGCGAAGA	1,722	-	*DnaJ*
Tt31c01g0005961	TTGAATGACACTTCGGTATG	GTTGCGTAACAGCGATTA	2,372	-	*GH*
Tt31c01g0005981	TTGCTTGCTTCCAGTCCT	CTCCACTCACAACATCCAA	881	-	*GH*
Tt31c02g0018691	CTCAACGCCATGCTCTTC	CAGCTAGTGGACGTGATG	1,187	-	*GH*
Tt31c01g0005601	GAGAGTGGTCAAGGAAGTG	GAGCCGCAATATCCAACA	940	1,426	Three GC-AG introns
Tt31c06g0056181	CCGTTATGGTGTGGAGTG	CGACAGTGGTGAAGACAG	679	802	GC-AG intron in non-coding regions
Tt31c01g0015591	ACACTCGTATATCTGCTGAA	CACCTGAATGCGTATTGC	716	1,021	One AT-AC intron and one GC-AG intron
Tt31c05g0047641	CGTTGTTGAGAACTGTATGG	CTGTTGGAGATTGAGGAGAA	757	1,485	One GT-AA intron and two GC-AG introns
Tt31c05g0047741	GACGATTGCTGCTGGTAT	AGAACGACGAGGAGTGAT	320	568	One GT-GT intron
Tt31c06g0057221	GGACGATGGATTGCGATA	CTGTGACTGGAGTGATATGT	605	2,203	One GA-AC intron

Note: “-” represents none.

Supplementary Table S3 Primers for detecting genetic variations between allelic *CtDnaJ* genes

Alleles	Forward primer (5'-3')	Reverse primer (5'-3')	Length (bp)	SNP loci	Indel loci
Ct29DnaJ.1/Ct31DnaJ.1	GCTCTATCTATCTGTCCTCTC	CTCGCTTCTTGTCGTCTT	500/500	12	-
Ct29DnaJ.2/Ct31DnaJ.2	ACTTCTTCTCGACGTTCAC	ACATCTGCTTTCTGGCTAC	406/415	8	1
Ct29DnaJ.6/Ct31DnaJ.6	GTCTGTCGTGCCTATGTC	AACCTTGTAGTAGTCCTTCTG	334/336	4	2
Ct29DnaJ.7/Ct31DnaJ.7	TTCTGGCTGTTCTGGTAAC	CTCAAGTGAACTAACAAGGTC	208/203	-	1
Ct29DnaJ.11/Ct31DnaJ.11	GAGGAGCGGAAGAAGAAC	CGATGATGGATGCGAAGA	1,268/1,270	12	1
Ct29DnaJ.12/Ct31DnaJ.12	GAACATTGGATTGCTGGTAA	AATCTTCTCGCTGTCGTAA	655/644	7	2
Ct29DnaJ.28/Ct31DnaJ.28	CTTGGCTGTCTGTTATAGATG	ACTACGATGAGTGCGATAG	468/468	5	-

Note: The values on the left and right of "/" represent the amplification lengths in Ct001_29 and Ct001_31, respectively; "-" represents none; "SNP" represents single nucleotide polymorphism; "Indel" represents insertions and deletions.

Supplementary Table S4 Primer sequences for constructing yeast two-hybrid vectors

Genes	Primer sequence (5'-3')
*CtDnaJ.16*_F	ggaggacctgcatatggccatggaggccATGTTCTCCTATGTCCTCGGCTC
*CtDnaJ.16*_R	ggttatgctagttatgcggccgcCTACTTCTTCACGAGGCCTTCC
*CtHSP70.1*_F	agattacgctcatatggccatggaggccATGACCAAGGCGATCGGTATT
*CtHSP70.1*_R	ccgtatcgatgcccacccgggTCAGTCGACCTCCTCGACCG
*CtHSP70.2*_F	agattacgctcatatggccatggaggccATGTCTCTTCCTCGTCGACCG
*CtHSP70.2*_R	ccgtatcgatgcccacccgggTCACAGCTCGTCGTGCGAG
*CtHSP70.3*_F	agattacgctcatatggccatggaggccATGAAACCCGGCCAGCCG
*CtHSP70.3*_R	ccgtatcgatgcccacccgggCTACAGCTCTTCATGTCTATGCGG
*CtHSP70.4*_F	agattacgctcatatggccatggaggccATGTTTACTGCTGCTCGTTCCC
*CtHSP70.4*_R	ccgtatcgatgcccacccgggTCAATCCTTCTTCTCCTCCTTGTT
*CtHSP70.5*_F	gtaccagattacgctcatatgATGTCCGTCGTCGGTATCGA
*CtHSP70.5*_R	cagctcgagctcgatggatccCTAGTCCACGTCCATCTCCGTC
*CtHSP70.6*_F	agattacgctcatatggccatggaggccATGAGTGTCGTGCTGCTCAGAG
*CtHSP70.6*_R	ccgtatcgatgcccacccgggTTATAGCTCAATGTGCGGGTAATAG
*CtHSP70.7*_F	agattacgctcatatggccatggaggccATGGAGGAAATCTTTGACGGTG
*CtHSP70.7*_R	ccgtatcgatgcccacccgggTCACCGCATGCCTGCGGT

Note: Lowercase letters represent homologous arm sequences, uppercase letters represent gene-specific sequences, and red text indicates restriction enzyme sites.

Supplementary Table S5 BUSCO assessment of Ct001_31 genome

Contents		Fungi_odb10	Agaricomycetes_odb10	Polyporales_odb10
Total		758	2898	4464
Complete		756 (99.74%)	2891 (99.76%)	4445 (99.57%)
	Single copy	750 (98.94%)	2869 (99.00%)	4403 (98.63%)
	Duplicated	6 (0.79%)	22 (0.76%)	42 (0.94%)
Fragment		2 (0.26%)	2 (0.07%)	1 (0.02%)
Missing		0 (0%)	5 (0.17%)	18 (0.40%)

Supplementary Table S6 Statistical analysis of RNA-Seq data obtained in this study

Sample	Ct001_29_25°C_1	Ct001_29_25°C_2	Ct001_29_25°C_3	Ct001_29_35°C_1	Ct001_29_35°C_2	Ct001_29_35°C_3	Ct001_29_35°C_4
Raw_Reads_Number	49,402,360	45,339,258	48,299,168	49,097,266	46,125,048	49,320,404	48,921,450
Raw_Bases_Number	7,410,354,000	6,800,888,700	7,244,875,200	7,364,589,900	6,918,757,200	7,398,060,600	7,338,217,500
Clean_Reads_Number	47,967,610	44,392,710	46,600,224	47,715,556	44,860,292	47,904,380	47,398,588
Clean_Reads_Rate (%)	97.1	97.91	96.48	97.19	97.26	97.13	96.89
Clean_Bases_Number	7,195,141,500	6,658,906,500	6,990,033,600	7,157,333,400	6,729,043,800	7,185,657,000	7,109,788,200
Low-quality_Reads_Number	0	0	0	0	0	0	0
Low-quality_Reads_Rate (%)	0	0	0	0	0	0	0
Ns_Reads_Number	41,376	27,996	31,218	31,538	32,268	31,952	28,250
Ns_Reads_Rate(%)	0.08	0.06	0.07	0.06	0.07	0.07	0.06
Adapter_Polluted_Reads_Number	1,393,374	918,552	1,667,726	1,350,172	1,232,488	1,384,072	1,494,612
Adapter_Polluted_Reads_Rate (%)	2.82	2.03	3.45	2.75	2.67	2.81	3.06
Raw_Q30_Bases_Rate (%)	89	88.53	89.57	89.18	89.31	89.43	89.6
Clean_Q30_Bases_Rate (%)	88.89	88.45	89.44	89.08	89.22	89.32	89.49

Sample	Ct001_31_25°C_1	Ct001_31_25°C_2	Ct001_31_25°C_3	Ct001_31_35°C_1	Ct001_31_35°C_2	Ct001_31_35°C_3
Raw_Reads_Number	45,504,188	48,703,126	50,055,550	48,990,876	48,425,228	49,709,604
Raw_Bases_Number	6,825,628,200	7,305,468,900	7,508,332,500	7,348,631,400	7,263,784,200	7,456,440,600
Clean_Reads_Number	44,163,650	47,183,556	47,428,144	47,355,810	45,735,370	46,902,146
Clean_Reads_Rate (%)	97.05	96.88	94.75	96.66	94.44	94.35
Clean_Bases_Number	6,624,547,500	7,077,533,400	7,114,221,600	7,103,371,500	6,860,305,500	7,035,321,900
Low-quality_Reads_Number	0	0	0	0	0	0
Low-quality_Reads_Rate (%)	0	0	0	0	0	0
Ns_Reads_Number	267,332	39,096	41,394	35,736	26,274	26,830
Ns_Reads_Rate (%)	0.59	0.08	0.08	0.07	0.05	0.05
Adapter_Polluted_Reads_Number	1,073,206	1,480,474	2,586,012	1,599,330	2,663,584	2,780,628
Adapter_Polluted_Reads_Rate (%)	2.36	3.04	5.17	3.27	5.5	5.59
Raw_Q30_Bases_Rate (%)	89.79	89.44	91.05	90.06	91.24	90.95
Clean_Q30_Bases_Rate (%)	89.76	89.33	90.92	89.97	91.1	90.79

Supplementary Table S7 Genes assigned to the GO terms “protein folding” and “response to heat”.

Gene number	GO term “protein folding”	GO term “response to heat”	Annotation
Tt31c09g0070281	√	√	HSP20
Tt31c09g0067631	√	√	HSP20
Tt31c01g0010311	√	√	ClpB (AAA+ family)
Tt31c05g0047881	√	√	ClpB (AAA+ family)
Tt31c09g0070341	√	√	HSP20
Tt31c28g0112961	√	√	HSP60
Tt31c09g0070401	√	√	HSP20
Tt31c28g0118361	√	√	HSP90
Tt31c28g0117001	√	√	Activator of Hsp90 ATPase
Tt31c09g0069961	√	√	HSP20
Tt31c09g0067211	√	√	HSP20
Tt31c09g0067691	√	√	HSP20
Tt31c09g0070761	√	√	HSP20
Tt31c09g0070651	√	√	HSP20
Tt31c34g0126781	√	×	HSP10
Tt31c07g0060661	√	×	DnaJ
Tt31c07g0057551	√	×	Tubulin binding cofactor C-domain-containing protein
Tt31c23g0104541a	√	×	Peptidyl-prolyl cis-trans isomerases
Tt31c01g0010071	√	×	SGS-domain-containing protein
Tt31c38g0136001a	√	×	Thioredoxin_13
Tt31c38g0130321a	√	×	Pkinase
Tt31c06g0048191	√	×	Golgi phosphoprotein 3

Supplementary Table S8 Information of allelic *CtDnaJ* genes

Gene	Amino acid	Theoretical isoelectric point	Molecular weight (kDa)	Gravity
CtDnaJ.1	504/504	9.27/9.22	53.91/53.88	-0.610/-0.609
CtDnaJ.2	222/225	8.64/7.82	23.99/24.24	-0.292/-0.296
CtDnaJ.3	153/153	4.52/4.52	16.75/16.72	-0.305/-0.310
CtDnaJ.4	656/656	5.43/5.43	72.99/73.01	-0.330/-0.329
CtDnaJ.5	375/375	6.54/6.97	41.89/41.91	-0.763/-0.772
CtDnaJ.6	547/547	8.73/8.48	60.23/60.25	-0.400/-0.389
CtDnaJ.7	324/324	9.69/9.69	35.06/35.04	-0.285/-0.285
CtDnaJ.8	495/495	9.39/9.13	55.31/55.37	-1.079/-1.087
CtDnaJ.9	217/217	7.96/7.96	25.40/25.40	-0.980/-0.980
CtDnaJ.10	445/445	8.96/8.96	47.90/47.90	-0.581/-0.581
CtDnaJ.11	528/528	6.48/6.48	57.57/57.62	-0.676/-0.671
CtDnaJ.12	323/323	6.41/5.94	35.95/35.87	-0.625/-0.614
CtDnaJ.13	608/613	6.35/6.46	68.26/68.68	-0.332/-0.327
CtDnaJ.14	227/227	10.27/10.34	25.87/25.85	-0.941/-0.950
CtDnaJ.15	550/550	5.43/5.38	62.24/62.22	-1.055/-1.051
CtDnaJ.16	332/332	6.98/8.25	37.60/37.66	-0.368/-0.397
CtDnaJ.17	378/378	9.10/9.10	40.03/40.03	-0.582/-0.584
CtDnaJ.18	670/538	9.62/9.19	75.23/59.87	-0.998/-1.085
CtDnaJ.19	221/221	9.43/9.43	25.31/25.32	-0.968/-0.968
CtDnaJ.20	374/374	6.32/6.32	41.62/41.62	-0.556/-0.556
CtDnaJ.21	415/415	5.01/5.01	45.46/45.46	-0.642/-0.642
CtDnaJ.22	572/572	6.17/6.29	62.27/62.24	-0.624/-0.611
CtDnaJ.23	452/452	9.20/9.20	50.30/50.35	-0.179/-0.190
CtDnaJ.24	226/226	10.10/10.10	25.15/25.14	-0.977/-1.000
CtDnaJ.25	390/390	8.86/8.86	44.56/44.59	-1.107/-1.101
CtDnaJ.26	229/229	10.09/10.19	25.59/25.69	-0.699/-0.717
CtDnaJ.27	366/366	5.44/5.61	40.80/40.84	-1.076/-1.045
CtDnaJ.28	398/398	5.83/5.83	44.13/44.11	-0.676/-0.675
CtDnaJ.29	483/483	6.60/6.43	53.60/53.59	-0.641/-0.640
CtDnaJ.30	573/573	6.45/6.37	64.62/64.56	-1.174/-1.183

Supplementary Table S9 Statistical analysis of genetic variations between allelic *CtDnaJ* genes

Alleles	SNPs	Indels	Length of Indels (bp)
Ct29DnaJ.1/Ct31DnaJ.1	16	-	-
Ct29DnaJ.2/Ct31DnaJ.2	17	2	2/9
Ct29DnaJ.3/Ct31DnaJ.3	4	-	-
Ct29DnaJ.4/Ct31DnaJ.4	24	-	-
Ct29DnaJ.5/Ct31DnaJ.5	18	2	3/2
Ct29DnaJ.6/Ct31DnaJ.6	21	3	7/1/1
Ct29DnaJ.7/Ct31DnaJ.7	9	1	5
Ct29DnaJ.8/Ct31DnaJ.8	12	-	-
Ct29DnaJ.9/Ct31DnaJ.9	15	-	-
Ct29DnaJ.10/Ct31DnaJ.10	1	-	-
Ct29DnaJ.11/Ct31DnaJ.11	25	1	2
Ct29DnaJ.12/Ct31DnaJ.12	20	2	10/1
Ct29DnaJ.13/Ct31DnaJ.13	9	-	-
Ct29DnaJ.14/Ct31DnaJ.14	2	1	10
Ct29DnaJ.15/Ct31DnaJ.15	16	-	-
Ct29DnaJ.16/Ct31DnaJ.16	3	-	-
Ct29DnaJ.17/Ct31DnaJ.17	9	-	-
Ct29DnaJ.18/Ct31DnaJ.18	11	-	-
Ct29DnaJ.19/Ct31DnaJ.19	15	-	-
Ct29DnaJ.20/Ct31DnaJ.20	2	-	-
Ct29DnaJ.21/Ct31DnaJ.21	-	-	-
Ct29DnaJ.22/Ct31DnaJ.22	23	1	8
Ct29DnaJ.23/Ct31DnaJ.23	29	1	4
Ct29DnaJ.24/Ct31DnaJ.24	6	-	-
Ct29DnaJ.25/Ct31DnaJ.25	12	-	-
Ct29DnaJ.26/Ct31DnaJ.26	8	-	-
Ct29DnaJ.27/Ct31DnaJ.27	16	-	-
Ct29DnaJ.28/Ct31DnaJ.28	9	-	-
Ct29DnaJ.29/Ct31DnaJ.29	10	1	2
Ct29DnaJ.30/Ct31DnaJ.30	7	-	-

Note: "-" represents none; "SNPs" represents single nucleotide polymorphisms; "Indels" represents insertions and deletions.
